# Genome sequence and genetic diversity analysis of an under-domesticated orphan crop, white fonio (*Digitaria exilis*)

**DOI:** 10.1093/gigascience/giab013

**Published:** 2021-03-12

**Authors:** Xuewen Wang, Shiyu Chen, Xiao Ma, Anna E J Yssel, Srinivasa R Chaluvadi, Matthew S Johnson, Prakash Gangashetty, Falalou Hamidou, Moussa D Sanogo, Arthur Zwaenepoel, Jason Wallace, Yves de Peer, Jeffrey L Bennetzen, Allen Van Deynze

**Affiliations:** Department of Genetics, University of Georgia, Athens, GA 30602, USA; Department of Plant Sciences, Seed Biotechnology Center, University of California, 1 Shields Ave. Davis, CA 95616, USA; Bioinformatics & Systems Biology, VIB / Ghent University, Technologiepark 71, 9052 Zwijnaarde, Belgium; Centre for Microbial Ecology and Genomics, Department of Biochemistry, Genetics and Microbiology, University of Pretoria, Pretoria 0028, South Africa; Centre for Bioinformatics and Computational Biology, Department of Biochemistry, Genetics and Microbiology, University of Pretoria, Pretoria 0028, South Africa; Department of Genetics, University of Georgia, Athens, GA 30602, USA; Institute of Plant Breeding, Genetics, and Genomics, University of Georgia, 111 Riverbend Rd, Athens, GA 30602, USA; International Crops Research Institute for the Semi-Arid Tropics (ICRISAT), BP 12404, Niamey, Niger; International Crops Research Institute for the Semi-Arid Tropics (ICRISAT), BP 12404, Niamey, Niger; Institut d'Economie Rurale, Ministere de l'Agriculture, Cinzana, BP 214, Ségou, Mali; Bioinformatics & Systems Biology, VIB / Ghent University, Technologiepark 71, 9052 Zwijnaarde, Belgium; Department of Crop and Soil Sciences, University of Georgia, 3111 Carlton St Bldg, Athens, GA 30602, USA; Bioinformatics & Systems Biology, VIB / Ghent University, Technologiepark 71, 9052 Zwijnaarde, Belgium; Centre for Microbial Ecology and Genomics, Department of Biochemistry, Genetics and Microbiology, University of Pretoria, Pretoria 0028, South Africa; College of Horticulture, Nanjing Agricultural University, Nanjing, China; Department of Genetics, University of Georgia, Athens, GA 30602, USA; Department of Plant Sciences, Seed Biotechnology Center, University of California, 1 Shields Ave. Davis, CA 95616, USA

**Keywords:** domestication, gene amplification, gene loss, millet, polyploidy

## Abstract

**Background:**

*Digitaria exilis*, white fonio, is a minor but vital crop of West Africa that is valued for its resilience in hot, dry, and low-fertility environments and for the exceptional quality of its grain for human nutrition. Its success is hindered, however, by a low degree of plant breeding and improvement.

**Findings:**

We sequenced the fonio genome with long-read SMRT-cell technology, yielding a ∼761 Mb assembly in 3,329 contigs (N50, 1.73 Mb; L50, 126). The assembly approaches a high level of completion, with a BUSCO score of >99%. The fonio genome was found to be a tetraploid, with most of the genome retained as homoeologous duplications that differ overall by ∼4.3%, neglecting indels. The 2 genomes within fonio were found to have begun their independent divergence ∼3.1 million years ago. The repeat content (>49%) is fairly standard for a grass genome of this size, but the ratio of *Gypsy* to *Copia* long terminal repeat retrotransposons (∼6.7) was found to be exceptionally high. Several genes related to future improvement of the crop were identified including shattering, plant height, and grain size. Analysis of fonio population genetics, primarily in Mali, indicated that the crop has extensive genetic diversity that is largely partitioned across a north-south gradient coinciding with the Sahel and Sudan grassland domains.

**Conclusions:**

We provide a high-quality assembly, annotation, and diversity analysis for a vital African crop. The availability of this information should empower future research into further domestication and improvement of fonio.

## Data Description

### Background

White fonio (*Digitaria exilis*, NCBI:txid1010633) is a vital cereal crop of West Africa, where it is commonly known as fonio or acha. A related *Digitaria* species, black fonio (*Digitaria iburura*), is a very minor crop, mostly of Nigeria, Benin, and Togo. Fonio (*D. exilis*) has an exceptionally small but very nutritious grain, with both high protein and high dietary fiber content [1–3]. Fonio can mature in as little as 8 weeks after planting and is commonly grown without fertilizer or irrigation on poor-quality soils in dry regions of the Sudan grasslands and Sahel. Although yields are low, the West African crop is harvested in early summer, where it fills a vital dietary gap before the maturation of sorghum or pearl millet crops in the same region. Perhaps no other crop deserves the title “orphan” more, because research attention on fonio has been minimal [4].

Wild *D. exilis* (sometimes called “hungry rice”) and other West African *Digitaria* have been harvested by farmers in times of famine throughout recorded history [4], but very little improvement has been made to the domesticated crop, at least partly evidenced by the fact that no controlled cross between fonio varieties has been substantiated. Fonio was probably domesticated in West Africa, presumably before the arrival of pearl millet or sorghum from Central and East Africa [5], as is suggested by the importance of fonio in Dogon and other creation myths [4]. Applying the term “domesticated” to fonio cultivars is, however, something of a stretch. Fonio cultivars do not exhibit the full set of domestication traits, in that they exhibit the shattering (grain release at maturity) and day-length dependence traits that have been selected against by early farmers across virtually all cereal crops [6, 7]. The selected mutations to non-shattering and day-length independence are routinely recessive, so the absence of these agricultural improvements may be an outcome of the polyploid nature of the fonio genome [8]. As an orphan crop, fonio has received very little research attention. Over the past 20 years, for instance, only 9 refereed publications report any new investigation of any aspect of fonio biology, although an additional ≥30 publications in that period investigated fonio agronomy, cultural significance, or nutritional properties [9, 10]. In 2007, Adoukonou-Sagbadja and colleagues [11] published a DNA marker-based analysis of fonio genetic diversity, and there are some transcript sequence data at NCBI [12]. Beyond this, most fonio investigations have been conducted in West Africa to determine appropriate conditions for subsistence farmers to grow and/or process the grain from local landraces. In contrast, several other orphan cereal crops of Africa and Asia have begun to receive extensive attention, including comprehensive analyses of germplasm resources, even to the extent of full genome sequence analysis. Three of these cereals with relatively deep recent analyses are, like fonio, panicoid grasses: foxtail millet (*Setaria italica*), pearl millet (*Cenchrus americanus*), and proso millet (*Panicum miliaceum*) [13–15]. With these panicoid grass resources, and a comparative genomics strategy [16], it should be possible to rapidly elevate fonio research to benefit fonio consumers and producers. This article describes our genomic sequence analysis of the fonio landrace Niatia, and a genetic comparison of fonio germplasms from across West Africa.

### Plant material and nucleic acid preparation

Fonio millet (cv. Niatia) seed was obtained from Dr. Sara Patterson (University of Wisconsin, Madison, WI, USA), which was collected in Mali at GPS coordinates 3.9861 W, 17.5739 N. Niatia is a popular local variety in Mali [17] (see Genetic Diversity for Nagoya protocol and germplasm access). The seeds were multiplied in a University of Georgia greenhouse. Seeds collected from a single plant were used for all DNA isolation. The seeds were surface sterilized with 8% sodium hypochlorite (Bioworld, Visalia, CA, USA) for 10 min, followed by 3 rinses with sterile distilled water. The plants were grown in standard potting soil (Fafard® 4M Sungro Professional Growing Mix, Sungro Horticulture, McClellan Park, CA, USA) in a greenhouse (with 14 h daylight and day/night temperatures of 26/20°C). They were watered daily to ∼70% soil water-holding capacity. The leaves of 4-week-old plants were used for DNA isolation, using a previously described protocol [18]. Briefly, leaf tissue (2.5 g) was ground in liquid nitrogen. After lysing in 15 mL of 2X extraction buffer (100 mM Tris–HCl pH 8.0, 1.4 M NaCl, 20 mM EDTA, 2% w/v CTAB with 10 μL/mL β-mercaptoethanol) and extraction with chloroform/isoamyl alcohol twice, the aqueous phase was then transferred to 3–3.5 volumes of precipitation buffer (50 mM Tris–HCl pH 8.0, 10 mM EDTA, 1% w/v CTAB). The sample was incubated overnight at room temperature to precipitate the DNA. After centrifugation at 3,500 rpm for 15 min, the DNA pellet was washed with ddH_2_O and centrifuged for 10 min. Then, 5 mL of 1.5 M NaCl and 6 μL of 10 mg/mL RNaseA was added to the pellet and incubated at 37°C until completely resuspended. A chloroform extraction was performed as above to remove RNaseA and any additional contaminants. The aqueous phase was collected and DNA was precipitated and washed with ethanol. The pellet was then resuspended in 100 μL ddH_2_O.

### PacBio SMRT sequencing, sequence polishing, and genome assembly

DNA samples were used to construct a PacBio (Pacific Biosciences, Menlo Park, CA, USA) SMRT sequencing library according to manufacturer recommendations at the University of California at Davis Genome Center. Fragments >10 kb were selected for sequencing via BluePippen (Sage Science, LLC, Beverly, MA, USA). A total of 88 Gb of raw PacBio reads from 76 SMRT cells were passed through the secondary analysis pipeline in SMRT Link (v6.0 [19]) and filtered for read quality >0.75 and length >1 kb. The resultant 75 Gb of filtered reads were assembled in Canu v1.8 (Canu, RRID:SCR_015880) [20] with the default settings for raw PacBio reads.

Racon (Racon, RRID:SCR_017642) was used to polish the original assembly for 2 rounds with the Canu-corrected PacBio reads. Sequentially, Arrow (VariantCaller v2.3.3) and Pilon v1.23 (Pilon, RRID:SCR_014731) were used to further polish the assembly with 36 Gb of Illumina paired-end reads obtained on the HiSeq 4000 (Illumina HiSeq 3000/HiSeq 4000 System, RRID:SCR_016386) at the Georgia Genomics and Bioinformatics Core at the University of Georgia.

The final assembly (Niatia v1.0) has a total length of 760.66 Mb and 3,329 contigs, with N50 of 1.73 Mb (L50 of 126) and N90 of 75.85 kb (L90 of 889). The longest contig is 10.17 Mb and the shortest contig is 1,013 bp, with a mean of 228.5 kb. We compare the quality of our genome with that of CM05836 [21], which was assembled using short reads, linked reads, and Hi-C. Although scaffold size is larger for the aforementioned genome, our genome has much better contiguity than CM05836 [21] as seen by N50 (1,734 vs 78 kb) and L50 (8 vs 2,624) (Supplementary Table 1). Scaffolding is expected to be higher in the latter genome because Hi-C technology was used that associates contigs on the same histone protein regardless of their size, but the Niatia genome shows much greater contiguity. To see the high contiguity in our genome assembly in detail, we took 2 of our medium-sized contigs (tig00001331 and tig00010942) as examples showing a dramatic improvement in contiguity in our genome, emphasizing the importance of long reads in assembly and annotation. This is further exemplified by comparing 2 random medium-sized contigs, tig00001331 corresponding to 100 consecutive segments anchored on the same chromosome 3B and tig00010942 corresponding to 65 consecutive segments on the chromosome 5A of the CM05836 [21] genome (Supplementary Fig. 1).

**Figure 1:**
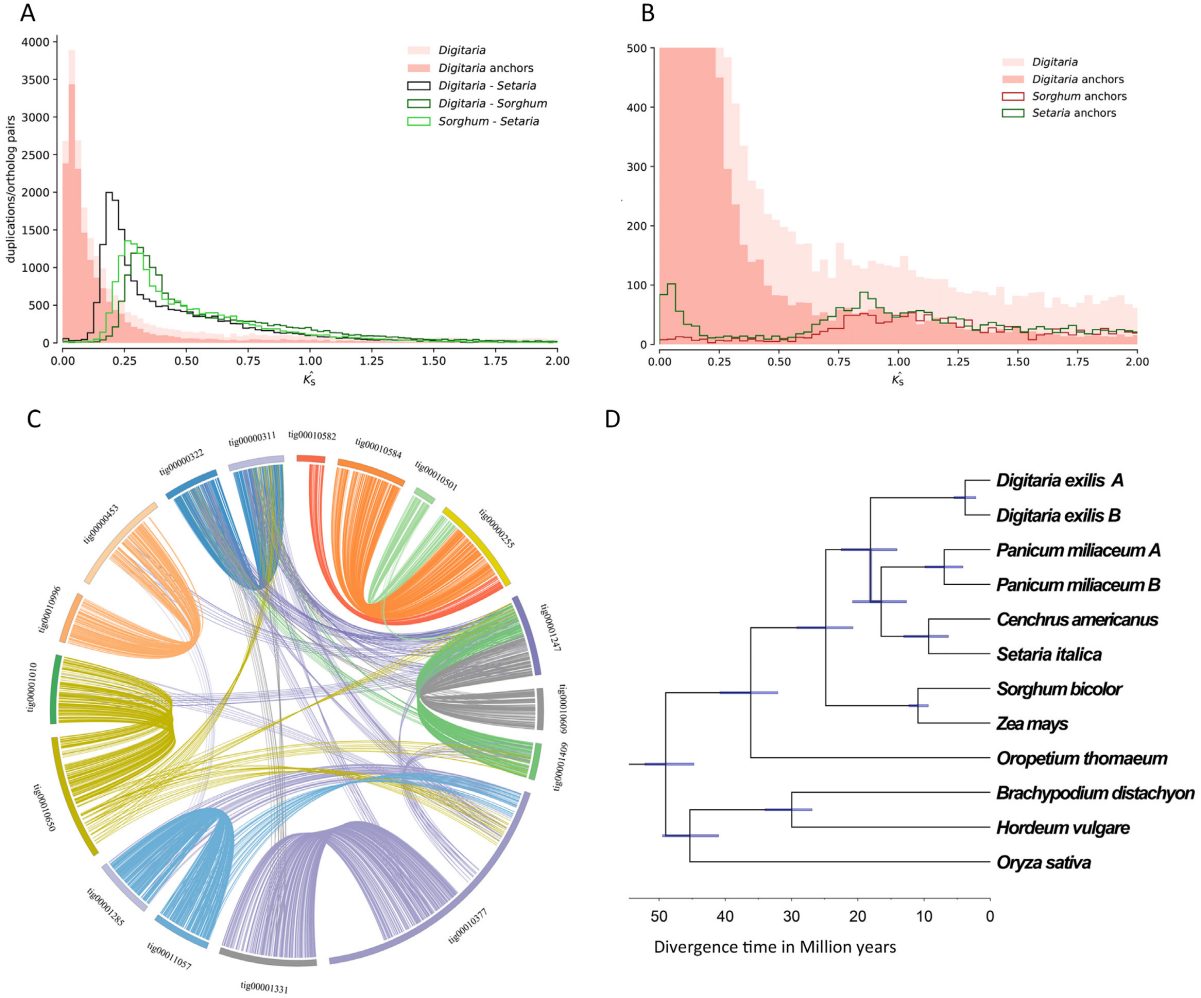
Whole-genome duplication and polyploidy analysis. (A) Ks estimation of age distribution for paralogs and orthologs of white fonio (*Digitaria*) and some close relatives. The distribution in light pink represents the entire white fonio paranome, while the distribution in darker pink represents the anchor points (duplicated genes lying in syntenic or collinear regions; see C). Distributions in black, dark green, and light green represent the 1-vs-1 ortholog comparisons between *Digitaria-Setaria, Digitaria-Sorghum*, and *Sorghum-Setaria*, respectively. (B) Ks distributions for paralogs of white fonio, sorghum, and *Setaria* (zoom in), showing an older, likely Poaceae-shared, WGD. (C) Syntenic relationships between putative homoeologous contigs, with colored lines connecting homoeologous gene pairs in the white fonio genome assembly. (D) Time-calibrated phylogenetic tree of several major Poaceae lineages, including white fonio, based on 1,242 gene families consisting of a single gene copy in each lineage and an anchor pair (A and B) in *Digitaria*. The time scale is shown in million years (My). See text for details.

**Table 1:** Summary of repeat sequence properties in the genome assembly

Class	Subclass	Type	No. of families	No. of repeats	Length (Mb)	Percent of genome
Class I TEs, retroelements	LTR-RT	Copia	353	45,194	22.8	3.0
		Gypsy	1,223	125,773	153.9	20.2
		Other	824	90,110	57.8	7.6
	LINE	I	17	3,040	1.5	0.2
	SINE		3,790	181,505	30.6	4.0
Class II TEs, DNA transposons	TIR	CACTA	348	42,737	7.4	1.0
		Mutator	34	8,493	1.8	0.2
		PIF	120	13,973	2.4	0.3
		Tc1	896	124,252	21.5	2.8
		hAT	93	13,097	2.5	0.3
	Helitron	Helitron	313	104,271	21.6	2.8
Tandem repeats	SSRs			133,570	5.9	0.8
Unclassified repeats	(Repbase)				48.0	6.3
	Total				329.8	49.7

### Estimation of genome size and heterozygosity

Kmer Analysis Toolkits [22] was used to count *k*-mers in Illumina raw reads and to compare the results with the *k*-mers counted from the genome assembly at several different *k*-mer sizes, from 17 to 30. These all yielded similar results but with a somewhat larger fonio genome predicted at smaller *k*-mer lengths. The distribution of *k*-mer counts was modeled and the heterozygosity level was estimated using GenomeScope2.0 [23].

Two distinct peaks were observed in the raw read *k*-mer distribution. We interpret the peaks at ∼50 and ∼100 counts/coverage as the 2 subgenomes in fonio (Supplementary Fig. S2). Genome size estimated from the peaks was 668–707 Mb, depending on the *k*-mer size used. This range of values is low compared to previous results from flow cytometry that indicated a genome size range of 830–1,000 Mb for a broad selection of *D. exilis* germplasm [4]. The underestimate is likely due to polyploidy confounding duplicated genes both within and among subgenomes. Single-nucleotide variation was estimated to be 4.3% when comparing the A and B genomes in this tetraploid, but slightly less than 0.01% heterozygosity was observed within either the A or B genomes, as assayed by *k*-mer allelic ratios. The *k*-mer counts in the assembled genome suggest that the peak at 100 counts represents common sequences between the 2 subgenomes, and the *k*-mers under the peak at 50 counts represent the divergent regions between the 2 subgenomes.

### Repeat annotation

Repeated sequences were mined and annotated with a combination of *de novo* and homology-based methods. First, simple sequence repeats (SSRs) were identified and masked with GMATA [24]. Long terminal repeat–retrotransposons (LTR-RTs) were identified *de novo* using the bioinformatic tools LTR_FINDER (LTR_Finder, RRID:SCR_015247) [25] and LTRharvest (LTRharvest, RRID:SCR_018970) [26], which use structural criteria to find intact LTR-RTs, followed by LTR_retriever analysis [27] to minimize false-positive results. SINE scan (version 1.1.1) [28] was used to find small interspersed nuclear elements (SINEs), a class of retroelements, and these were confirmed by manual investigation. Long interspersed nuclear elements (LINEs), another class of retroelements, were found with MGEscan-nonLTR (version 2) [29]. Small DNA transposable elements (TEs) were found with MITE Tracker [30], and HelitronScanner [31] was used to identify the DNA transposons called "Helitrons." All of the TEs from the genome assembly were used to generate a fonio-specific TE library, with individual TE families named according to the prevalent current nomenclature system [32]. The fonio TE library was compared to the Repbase [33] multispecies repeat repository to validate annotations and to discover any additional candidate repeats represented in Repbase. Then, the fonio TE library was used to identify both full-length and truncated TE elements by homologous search with RepeatMasker version 4.0.7 (RepeatMasker, RRID:SCR_012954) [34] in the genome assembly. Parameter settings were adopted from the analysis described in a previous publication [35]. The predicted insertion dates of intact LTR-RTs were calculated with LTR_retriever (LTR_retriever, RRID:SCR_017623) [27]. The SSRs and TEs were masked by Ns and a TE annotation file in GFF3 format was generated for subsequent gene annotation. Types and abundances of TEs and other repeats discovered in the fonio genome are presented in Table 1.

### Transcriptome assembly, candidate gene annotation, and BUSCO quality assessment

Illumina RNA sequencing data (paired-end 100 bp) of *D. exilis* were downloaded from the NCBI SRA (accession No. SRX1967865 [12]) from RNA consisting of ∼80% inflorescence and ∼20% leaf tissue. FastQC (FastQC, RRID:SCR_014583) [36] was used to evaluate data quality, and low-quality reads and adapter sequences were removed using Trimmomatic (Trimmomatic, RRID:SCR_011848) [37]. The remaining reads were aligned to the genome assembly using HISAT2 (HISAT2, RRID:SCR_015530) [38]. The spliced alignments were used as input for StringTie [39] and assembled into transcripts. TransDecoder, a companion software package of the Trinity platform [40], was used to predict open reading frames (ORFs).

For gene prediction and genome annotation, we used the Maker-P pipeline [41], in combination with Augustus (Augustus, RRID:SCR_008417) [42], SNAP [43], and GeneMark (GeneMark, RRID:SCR_011930) [44]. Augustus gene models came from the BUSCO (BUSCO, RRID:SCR_015008) [45] dataset identified during the assembly (see below). GeneMark_ES was used to produce *ab initio* gene predictions. Detailed settings for each round of Maker can be found in the Supplemental Methods. The first round of gene prediction with Maker used the following inputs: the RNAseq assembly described in the previous section, protein fasta sequences from *S. bicolor* and *S. italica* [46] as well as the repeat models for *Digitaria* (described above), and the soft-masked genome assembly. A second round of Maker used as input the genome file, the annotation produced by the previous round, and a SNAP species parameter/hmm file based on the prior annotation. Finally, the third round of Maker was run using the following input: the genome assembly, the annotation produced by round 2, and the GeneMark models. Functional annotation was done using the accessory scripts of Maker as described by Campbell and coworkers [47]. Briefly, a BLAST [48] search against the Swissprot database was used to assign putative functions to the newly annotated gene models, while InterProScan 5 (InterProScan, RRID:SCR_005829) [49] was used to obtain domain information.

Following mapping of RNAseq data with HISAT2, 88% of the RNAseq reads could be well aligned to the genome. Transcripts were assembled with Stringtie and ORFs were predicted with TransDecoder (TransDecoder, RRID:SCR_017647). A total of 58,305 candidate transcripts were obtained, of which 50,389 had predicted ORFs.

Our first round of Maker predicted 60,300 protein-coding genes (based only on RNA evidence and protein evidence from sorghum and *Setaria*). After the second and third round, where Augustus, SNAP, and Genemark-ES models were included, the number of predicted protein-coding genes increased to 67,921 and finally to 68,302. We removed 447 candidate genes that were judged to be spurious because they were fragments of otherwise fully assembled genes in the annotation, so the final number of genes annotated as protein-coding genes is 67,855. The statistics for the gene annotation can be found in Supplementary Table S2. In total, 88.3% of the gene models were supported by RNAseq data. The annotation edit distance (AED) measurements indicate how well an annotation agrees with overlapping evidence (protein, messenger RNA, or expressed sequence tag data). In the fonio assembly, >90% of the gene models have an AED score <0.4%, indicating that gene models are well supported by evidence. The number of genes and gene model lengths are greater than that reported by Abrouk et al. [21] for CM05836 (59,844), indicating the importance of long-read assemblies and contiguity in genome assembly and annotation.

BUSCO v 4.0.2 [45, 50] analysis of the filtered predicted protein sequences against the reference set for plants, on the gVolante platform [51], showed that 98.1% of the BUSCO genes were found as complete genes, while this representation number increased to 99.3% if partially covered BUSCO genes were added compared to the 97.2 reported by Abrouk et al. [21]. A total of 11.6% of the BUSCO genes were single copy, while 86.5% of the BUSCO genes were found in duplicate. Approximately 1.2% of the BUSCO genes were fragmented and ∼0.7% were missing.

A total of 4,741 non-coding RNAs (see Supplementary Table S3) were predicted with Infernal [52] by comparing the genome fasta file with the RFAM CM database, version 14.2 [53], using the protocol described by Kalvari et al. [54]. Most of these non-coding RNAs were found to be transfer RNAs (31.2%), 5S ribosomal RNAs (12.2%), and small nucleolar RNAs (23.4%), as seen in other plant genomes.

### Phylogenetic divergence and dating the most recent whole-genome duplication

The coding DNA sequences and annotations for *S. bicolor* and *S. italica* were downloaded from the PLAZA database [46]. Ks distribution analyses were performed using the wgd package (v1.1) [47]. For each species, the paranome (entire collection of duplicated genes) was obtained with “wgd mcl” using all-against-all BlastP [47] and MCL clustering [55]. Ks distributions were then constructed using “wgd ksd” with default settings (using MAFFT for multiple sequence alignment [56], codeml for maximum likelihood estimation of pairwise synonymous distances [57], and FastTree [FastTree, RRID:SCR_015501] [58] for inferring phylogenetic trees used in the node-weighting procedure). Anchors or anchor pairs (duplicates lying in collinear or syntenic regions of the genome) were obtained using i-ADHoRe [59] using the default settings in “wgd syn.”

We obtained gene families for a set of 9 species in the Poaceae family using OrthoFinder (OrthoFinder, RRID:SCR_017118) with default settings [60]. All sequence data were obtained from PLAZA [46]. From this set of gene families, we identified all gene families that were single-copy in all species but duplicated in *D. exilis*, and where the *D. exilis* duplicates were anchor pairs (1,967 gene families). For these gene families, we performed pre-alignment homology filtering using PREQUAL [61] and multiple sequence alignment of the masked amino acid sequences using MAFFT (MAFFT, RRID:SCR_011811) [56]. For each multiple sequence alignment, we obtained the corresponding codon-level nucleotide alignment. For each obtained nucleotide alignment, we sampled tree topologies from the posterior using MrBayes v3.2 (MrBayes, RRID:SCR_012067) [62] under the GTR model with a discrete Gamma mixture for relative substitution rates across sites (using 4 classes), sampling every 10 iterations, for a total of 250,000 iterations. We then identified all gene families for which the expected species tree topology had posterior probability >0.9, resulting in a set of 1,242 gene families. A concatenated codon alignment was obtained for these families, which was in 3 partitions corresponding to each codon position. We then performed posterior inference of substitution rates and divergence times for the partitioned alignment using MCMCTree [55, 63] using the multivariate normal (MVN) approximation of the likelihood (where the MVN approximation was based on the maximum likelihood estimates under the GTR model with Gamma distributed relative rates across sites [5 categories]). We used a Gamma (2, 11) prior for the mean substitution rate per site per 100 My (million years), based on a rough estimate of the substitution rate under the molecular clock with a root age of 50 My obtained using baseml from the PAML package [53]. We use an independent log-normal rates relaxed molecular clock prior on branch-specific substitution rates, using a Gamma (2, 10) prior for the variance parameter of the clock. We set the birth-death-sampling prior such that a uniform prior over node ages is obtained. We include 2 fossil calibrations. First, we used a minimum age for the *Oryza—Hordeum* divergence of 34 My based on the review of Iles et al. [64]. Next, a secondary calibration for the root based on previous dating studies included in the TimeTree [65] database was used, where we excluded all time estimates younger than the 34 My constraint and older than 80 My. We then fitted a log-normal distribution to the age estimates in the time tree data, which we approximated by a Gamma (47, 100) distribution. We used MCMCTree to obtain 5,000 from the posterior sampling every 200 iterations after a burn-in of 50,000 iterations. We compared 2 independent runs with each other to verify convergence and with a run of the MCMC algorithm under the prior alone to compare the posterior distribution for the node ages to the effective prior implied by the fossil calibrations (Supplementary Fig. S3). The results of this analysis provide the phylogenetic tree shown in Fig. 1D.

### Transposable element properties

The ∼42.6% TE content of the fonio genome is a minimal estimate, given that degraded TE fragments are often missed by the *de novo* discovery analysis that was used. This underestimation is routine in other plant genome annotations as well [66], so it is reasonable to compare TE descriptions across plant genomes. In fonio, the very high level of Gypsy LTR-RTs compared to Copia LTR-RTs is exceptional. Although most grass genomes have more Gypsy TEs than Copia (e.g., ∼50% Gypsy and ∼25% Copia in the ∼2.4-Gb maize genome [67] or ∼36% Gypsy and ∼33% Copia in the ∼2.8-Gb pearl millet genome [14]), the ∼6.7:1 Gypsy to Copia ratio in the ∼900-Mb fonio genome is unprecedented. One should remember, however, that the diploid constituent genomes of fonio are ∼450 Mb, so somewhat similar results are observed in other small panicoid genomes like sorghum (∼750 Mb) and rice (∼430 Mb), with Gypsy/Copia of ∼3.7 and ∼4.9, respectively [68]. This fonio observation is surprising because the quantity of Gypsy LTR-RTs is the major determinant of genome size in grasses [69], so one would expect higher Gypsy to Copia ratios as genome size increases, rather than the opposite that we observe. These results suggest that either different factors initiate Gypsy amplification bursts than Copia amplifications, or that Copia elements are particularly sensitive to shared activation factors. It would be useful to investigate additional *Digitaria* species to see whether this Gypsy/Copia ratio trait is shared by other close relatives and thus a possible outcome of common ancestral properties.

Analysis of LTR-RT insertion dates demonstrated that most of the elements had been inserted within the past 2 My. This high level of recent activity is a standard observation in the grasses, at least partly caused by the fact that the rapid DNA removal by accumulated small deletions quickly excises and otherwise obscures any DNA that is not under positive selection [70, 71].

### Whole-genome duplication and subsequent stability

We inferred whole-paranome and 1-vs-1 ortholog Ks distributions and performed syntenic analyses to further assess the clear signature of a relatively recent whole-genome duplication (WGD) in *Digitaria exilis*.Ks distributions present a clear signature of WGD in the recent evolutionary past of *D. exilis*, with this event not shared with the closest relative in our analyses (*S. italica)* (Fig. 1A). We note that a trace of an older, likely Poaceae-shared WGD [72] event was also clearly observed in both the whole-paranome and anchor pair Ks distributions of *D. exilis*, coinciding with similar signatures in sorghum and *Setaria* (Fig. 1B). Analysis of collinearity and synteny show that the genome of *D. exilis* is still largely conserved in duplicate (Fig. 1C). Phylogenetic divergence time estimation (Fig. 1D) estimated the timing of the WGD event (or divergence of parental genomes in the case of an allopolyploidy event) at ∼3.1 million years ago (mya) with a 95% posterior uncertainty interval of (2.2, 4.2 My) and the divergence of *Digitaria* from *Setaria* at 17.8 (12.5, 23.1) mya, with these estimates associated with a posterior mean substitution rate across the 3 codon positions of 2.5 × 10^−9^ (1.1 × 10^−9^, 5.0 × 10^−9^) substitutions per year per site. This is consistent with CM05836 [21]. The closest relatives to fonio with whole-genome sequences would be *P. miliaceum, S. italica*, and *C. americanus*. The diploid ancestor to *D. exilis* is not clear [73].

It is interesting that Fig. 1C shows extreme conservation of gene content and order across long scaffolds but also the presence of large rearrangements that differentiate chromosome-size blocks. This suggests a possible selection for major rearrangements after the polyploids were formed, perhaps to minimize tetrasomic inheritance [74, 75].

In the ∼3.1 My since the latest WGD, most of the duplicated genes have had both copies retained. For instance, the BUSCO gene set yielded 86.5% of the genes still in a duplicated state. Our genome assemblies did not yield complete chromosomes, so we could not investigate the details of major chromosomal rearrangements, preferential gene loss (also known as fractionation), or parent-specific gene expression differences that might differentiate the 2 ancestral genomes in this tetraploid [76]. The large stretches of gene content and gene collinearity retention observed between our largest contiguous assemblies (Fig. 1C) do demonstrate, however, that there has been no large number of small rearrangements of these genomes over the past 3.1 My.

### Expansions and contraction of gene families

To see the expansions and contractions of gene families, broomcorn millet (*Panicum miliaceum* L.) was added in the phylogenetic analysis because it experienced a recent tetraploidization estimated at ∼5.8 mya that is similar to fonio.

**Figure 2:**
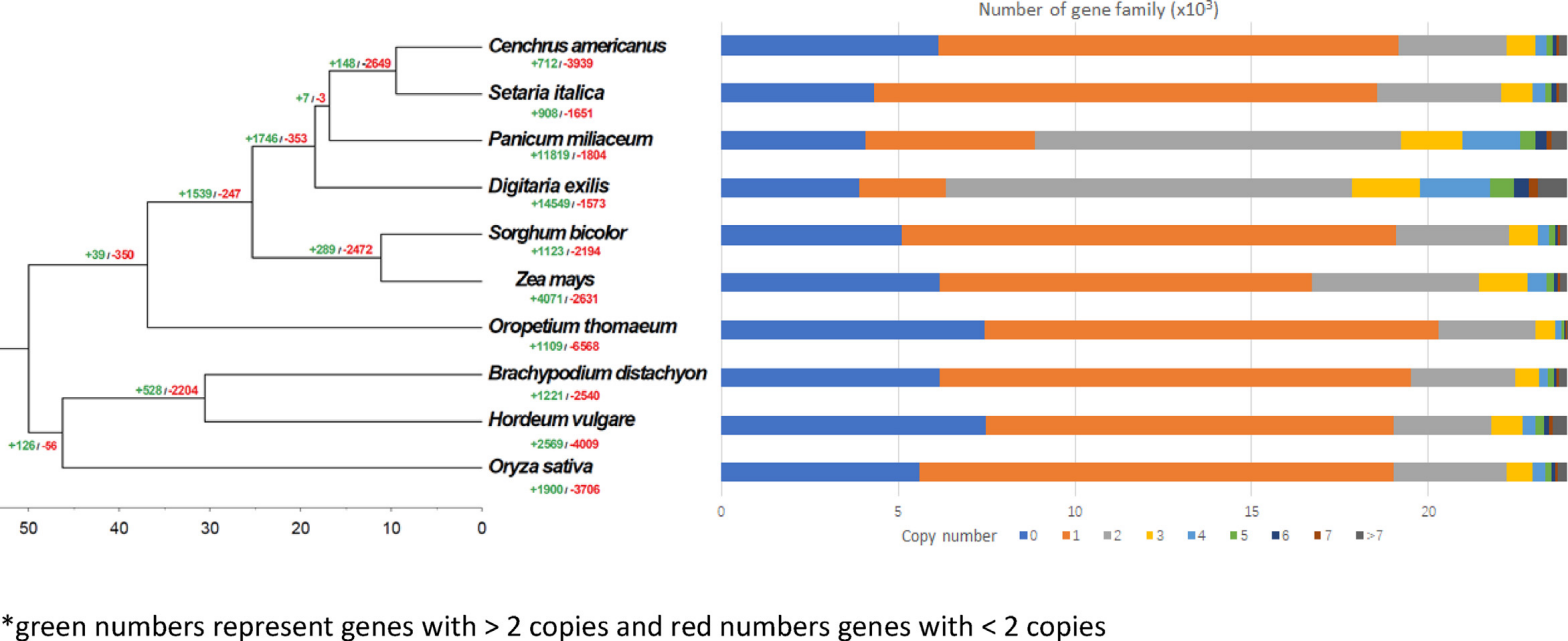
The number of gene families that expanded or contracted during evolution mapped to the species phylogenetic tree in related Poaceae species.

Based on sequence homology, we assigned 58,459 genes to 20,003 families, 14,549 of which have expanded in the fonio genome. Expansion in a similar number of gene families (11,819) was also observed in the broomcorn millet genome, also an allotetraploid crop. Of the fonio gene families, 57.4% contain 2 copies (the most abundant category in these 10 species) and 30.4% contain >2 copies (Fig. 2). Most (∼90%) of the 2-copy gene families of fonio are located in syntenic blocks, indicating that the expansion was mainly due to the recent WGD event (Fig. 2 and Supplementary Fig. S4).

In addition to the majority of multi-copy genes, there are many (∼12.1% of the total) that are single-copy genes and thus a likely outcome of at least some deletion after polyploidy. Gene Ontology enrichment analyses of contracted genes (1 copy; Supplementary Fig. S5) and expanded genes (>2 copies; Supplementary Fig. S6) relative to *O. sativa* were performed. The analysis identifies negative regulators and recognition factors for biotic and abiotic stresses, as well as pollen/fertility recognition, as single-copy genes. In contrast, there is general expansion of gene families encoding positive regulators of multiple-copy genes. These results suggest that further analysis of these genes may reveal their roles in heat and drought stress tolerance, and in understanding of crossing barriers in fonio.

### Candidate domestication genes

Improvement of fonio will require further domestication, particularly to solve the issues of shattering and lodging. This process should be greatly assisted by the provision of a comprehensive genome sequence.

In rice, sorghum, and maize, mutations in the gene *SSH1* (SUPPRESSION of SEED SHATTERING-1) are associated with panicle retention of the grain after seed maturation (the “non-shattering” trait) in domesticated accessions [77]. Nine sequenced grass genomes were scanned with OrthoFinder (as described in the section “Phylogenetic divergence and dating the most recent whole-genome duplication”) to find the orthologues of this gene. The gene family fasta files were used to construct trees using MAFFT and Iqtree, and trees were visualized in FigTree. Interproscan was used to annotate the proteins with their pFam domains, and alignments were visualized in Geneious Prime [78].

Fonio has 4 genes related to *SSH1*, but the phylogenetic tree indicated that 2 are more closely related to the rice *SSH1* gene associated with shattering than to the other *SSH1*-like gene in rice (Supplementary Fig. S7). Other species included in our dataset have between 1 and 3 *SSH1*-like genes (Supplementary Table S4). The extra copies in *D. exilis* are expected because of its polyploid nature and thus can explain why no ancient or modern farmers have detected recessive single-gene mutations at each of these loci in a single fonio plant. By modern forward or reverse genetic and breeding techniques, inactivation and selection of both of these genes should be targeted to solve the shattering problem in fonio.

Inactivation of the *dw3* (Dwarfing-3) genes of sorghum is responsible for the semi-dwarf trait that diminishes lodging and thereby greatly improves yield and input response in this important crop of arid and semi-arid agriculture [79]. Inactivation-mutant orthologues of the same gene are also responsible for the pearl millet cultivars with highest lodging resistance and the highest grain yield [80]. Hence, orthologues of *dw3* also should be targets for inactivation-mutation and molecular breeding in fonio. Once again, fonio has more copies of this gene than do any of the other grasses screened, all of which are diploids (Supplementary Fig. S8 and Table S5).

The *GW2* (GRAIN WEIGHT-2) gene controls seed weight in wheat and rice, with inactivation of the gene leading to larger grain [81, 82]. Orthofinder results indicated that members of this gene family are present in single copy in all of the examined grass species, except fonio and maize (Supplementary Fig. S9 and Table S6). The 2 copies in *D. exilis* only differ from each other by 3 amino acid residue substitutions. The fonio genes were found to be nearly identical to the unmutated *GW2* version that yields smaller grain in rice and wheat (data not shown). Although increased seed weight does not always increase yield (due to correlated traits, such as seed number), it is a particularly important trait in fonio to enable sowing for uniform stands and mechanical threshing.

### Genetic diversity

Fonio genetic diversity was assessed using 184 samples from ∼130 accessions collected from Mali and Niger, signatories to the Cartagena Protocol on Biosafety (Supplementary Table S7). Consistent with the Nagoya Protocol and the third objective of the Convention on Biological Diversity of access and benefit sharing, fonio materials from Mali were collected in Mali by Institut d'Economie Rurale (IER) while those from Niger were collected in Niger by Institut National la de Recherche Agronomique du Niger (INRAN) and conserved at the ICRISAT Niamey genebank. M.D.S., F.H., and P.G. were involved in the germplasm collection, seed conservation at the genebank, and/or DNA extraction from young seedlings. All DNA samples or seed were sent to the USA for analysis for research purposes only. This research has no direct commercial application.

**Figure 3:**
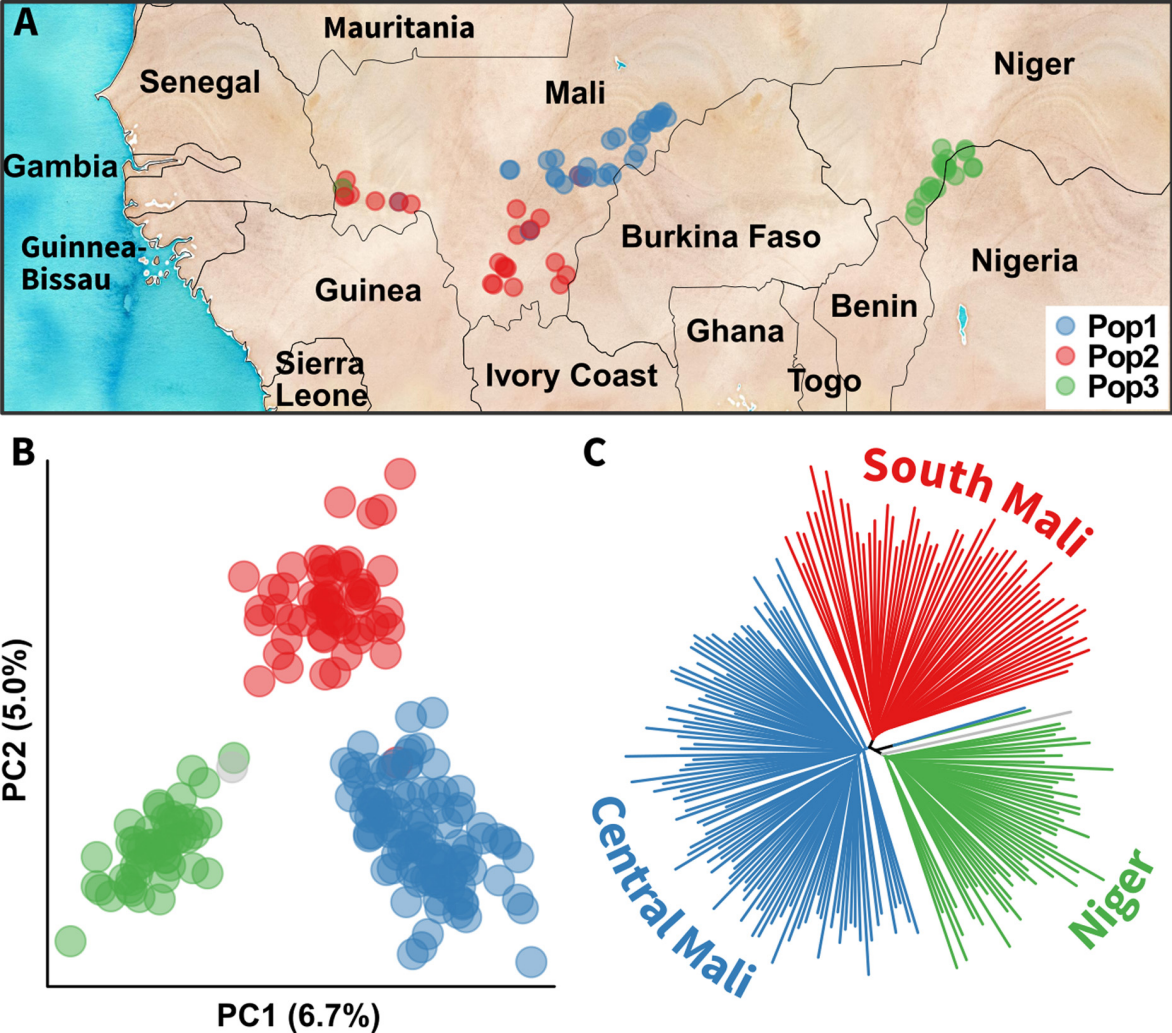
Fonio genetic diversity. The genetic diversity of fonio samples was surveyed by genotyping-by-sequencing. (A) Fonio samples originated from Mali and Niger. They separate into 3 primary subpopulations based on population structure analysis. Both principal coordinate analysis of the genetic diversity (B) and a neighbor-joining tree of the population (C) confirm these groupings. A few discrepancies between population assignment and geography may be due to recent long-distance germplasm exchanges or labeling errors during collection and storage.

Seedlings of each sample were grown at the respective institutions in West Africa, and DNA was extracted from young leaves with a QIAGEN DNeasy Plant Mini Kit (Germantown, MD, USA). Lyophilized DNA was then sent to Data2Bio (Ames, IA, USA) for tunable genotyping-by-sequencing using 2-bp selection and 5 runs on an Ion Torrent Ion Proton Instrument (Thermo Fisher Scientific, Waltham, MA, USA). The resulting raw sequences were quality-trimmed by Data2Bio, which removed bases with PHRED quality scores <15. These trimmed sequences were then aligned to the genome assembly with GSNAP v2020–04-08 [83] using default parameters. Single-nucleotide polymorphisms were called using the bcftools mpileup command v1.9 [84] with max-depth set to 1,000 and minimum base quality set to 20. Raw single-nucleotide polymorphisms were then filtered using TASSEL v5.2.40 [85], custom R scripts with R v3.5.1 [86], and bcftools to include only sites with ≤25% heterozygosity, ≤500 total read depth, ≤60% missing data, and ≥2.5% minor allele frequency (Supplementary Table S8). Population substructure was determined with fastStructure v1.0 [87], testing from 1 to 10 population clusters and identifying the optimal number with the included chooseK.py program. This identified 3 clear clusters of material, with genetic separation strongly correlated with geography (Fig. 3A). The genetic distinctions among these clusters are clear when plotting the genetic principal coordinates and relationship dendrogram (Fig. 3B). A small number of accessions (<5) appear “misplaced” on the geographic map, which could be due to recent transfer of germplasm or human error during collection, storage, or processing. Geographic clustering can reflect both human trafficking of seed stocks and the genetic basis of local adaptation. Further (both broader and deeper) germplasm analyses will be useful for resolving these issues.

## Conclusions

Genome analysis of any polyploid is challenging, especially when no diploid ancestors are known. Our sequence of the white fonio (*D. exilis*) genome indicates its recent tetraploid origin and the retention of most of the genes duplicated in this process. This retention of duplicated genes likely explains why recessive mutations for important agronomic traits like shattering, seed size, semi-dwarfism, and others like day-length dependence have not yet been detected in fonio. However, it is now possible to identify such mutations by using modern mutation detection schemes, like those used for the tetraploid cereal *Eragrostis tef* [88]. One purpose for generating a fonio genome sequence was to attract molecular genetics researchers to the study of this crop and thereby enable hypothesis-driven breeding through genomics-assisted selection. If future researchers develop a transformation technology for fonio [89] or develop other genome-editing strategies [90], then directed mutagenesis could be used to knock out pairs of these domestication genes in a single step [91].

The importance of correcting such problems as shattering, seed size, and lodging in fonio cannot be overestimated. Until shattering is solved, farmers will continue to be required to harvest before grains fully mature, thus dramatically decreasing overall yield. Without semi-dwarf varieties, already serious lodging problems in fonio will continue to prohibit the use of more inputs (because fertilizer increases plant height and thus lodging) or even the selection of larger grain yield from the panicles because greater weight on the top of the plant can cause more lodging. The same will almost certainly be true for fonio, hence providing a partial explanation for its tiny seed size in cultivated landraces. With domestication traits fully penetrant into fonio cultivars, one can expect dramatic increases in fonio performance, with expectations of a 2-fold or greater yield enhancement easily within the short-term range of possibilities.

The absence of an outcrossing protocol for fonio is another technical deficiency that severely limits this crop's potential for improvement. Our diversity analysis on cultivar Niatia indicates <0.01% heterozygosity, showing that crosses occur very rarely by natural processes. Hence, generating controlled crosses will probably require a serious dedication to this pursuit. Our results indicate a great deal of genetic variability within fonio landraces, so we have no doubt that hybridization could be used in breeding projects to optimize fonio germplasm quality for future West African and other farmers.

## Data Availability

The genome and annotation underlying this article are available in the African Orphan Crops Consortium–specific branch of the ORCAE platform [92, 93] athttps://bioinformatics.psb.ugent.be/orcae/aocc/overview/Digex. The GenBank project number for the assembly is PRJNA640067. All scripts for diversity analysis and data tables are available at [94] including full genotyping table. Genotyping table is also available at GenBank Project No. PRJNA644458. All supporting data and materials are available at the *GigaScience* GigaDB database [95].

## Additional Files

**Supplementary Methods**.

**Supplementary Figure S1**. A. Comparison of the contiguity of the Niatia Genome and CM05836 [21] genome. B. Comparison of contig tig00001331 corresponding to 100 consecutive segments anchored on the same chromosome 3B and tig00010942 corresponding to 65 consecutive segments on the chromosome 5A on the Abrouk et al. [21] genome.

**Supplementary Figure S2**. The *k*-mer distribution of raw Illumina reads at *k*-mer value 33 bp.

**Supplementary Figure S3**. A. Marginal posterior distributions for 2 independent chains (green and orange) and induced marginal prior distributions (blue) for internal node ages (t_n11 to t_n19, see panel C), overall mean substitution rate (mu), mean substitution rate for different codon positions (mu1, mu2, and mu3), and variance parameter of the uncorrelated relaxed clock (sigma2_1, sigma2_2, and sigma2_3) for the 3 codon positions. B. Trace plots for the MCMC chains associated with panel (A).

**Supplementary Figure S4**. There are 10,075 families that have 2 copies in fonio and 1 copy in *Setaria italica*, and 90% of 2-copy families are located in synteny blocks. The above 4 examples indicate the high degree of collinearity and synteny between *S. italica* and fonio.

**Supplementary Figure S5**. GO of single-copy, contracted genes in fonio.

**Supplementary Figure S6**. GO enrichment for expanded genes in *D. exilis*and relative to *O. sativa*.

**Supplementary Figure S7**. Phylogenetic tree of the SSH-like genes from fonio and related species. The genes shaded in light blue are the family members most closely related to *SSH-1* in *O. sativa* and *D. exilis*. Genes are named according to their PLAZA identifiers. Abbreviations for species names are as follows: Bradi (*Brachypodium distachyon*), pgl_GLEAN (*Cenchrus amercianus*), Digex (*Digitaria exilis*), Oropetium (*Oropetium thomaeum*), OsR (*Oryza sativa*), Seita (*Setaria italica*), Sobic (S*orghum bicolor*), and Zm (*Zea mays*).

**Supplementary Figure S8**. Phylogenetic tree of the*dw3* gene family of fonio and related species.

**Supplementary Figure S9**. Gene family tree for GW2-A-like genes in fonio and related species. This figure also includes the genes from 2 additional Pooid species, barley (*Hordeum vulgare*) (HORV) and wheat (*Triticum turgidum*) (TRITD).

**Supplementary Table S1**. Comparison of genome assembly statistics of fonio.

**Supplementary Table S2**. Statistics for the gene annotation.

**Supplementary Table S3**. Annotated non-coding RNA genes.

**Supplementary Table S4**. Orthologs for suppression of Shattering1 genes.

**Supplementary Table S5**. Orthologs of Dwarf Gene-3.

**Supplementary Table S6**. Orthologs of Grain Weight-2 genes.

**Supplementary Table S7**. Passport data for accessions and samples used for diversity study (see Supplementary Tables Excel file).

**Supplementary Table S8**. Single-nucleotide polymorphism database used for diversity study (see Supplementary Tables Excel file).

## Abbreviations

AED: annotation edit distance; BLAST: Basic Local Alignment Search Tool; BUSCO: Benchmarking Universal Single-Copy Orthologs; CTAB: cetyl trimethylammonium bromide; Dw3: dwarf3; EDTA: ethylenediaminetetraacetic acid; Gb: gigabase pairs; GO: Gene Ontogeny; GW2: grain weight2; kb: kilobase pairs; LINE: long interspersed nuclear element; LTR: long terminal repeat; LTR-RT: long terminal repeat retrotransposon; MAFFT: Multiple Alignment with Fast Fourier Transform; Mb: megabase pairs; MITE: miniature inverted repeat transposable element; My: million years; mya: million years ago; NCBI: National Center for Biotechnology Information; ORF: open reading frame; PacBio: Pacific Biosciences; PAML: Phylogenetic Analysis by Maximum Likelihood; SINE: small interspersed nuclear element; SMRT: single-molecule, real-time sequencing; SRA: Sequence Read Archive; SSH1: suppression of shattering1; SSR: simple sequence repeat; TE: transposable element; TIR: terminal inverted repeat transposable element; WGD: whole-genome duplication.

## Competing Interests

The authors declare that they have no competing interests.

## Funding

J.L.B. acknowledges the Giles Fellowship from the University of Georgia as a source of funding for this project. Y.V.d.P. acknowledges funding from the European Research Council (ERC) under the European Union's Horizon 2020 research and innovation program (grant agreement No. 833,522). A.V. acknowledges funding from the Seed Biotechnology Center, University of California. J.G.W. acknowledges funding from the International Crops Research Institute for the Semi-Arid Tropics (ICRISAT) and the University of Georgia. M.D.S. acknowledges funding from the McKnight foundation.

## Authors' Contributions

J.L.B., J.W., Y.V.d.P., and A.V.D. conceived, designed, and interpreted the study; S.C., X.M., X.W., A.E.J.Y., S.R.C., M.S.J., P.G., F.H., M.D.S., and A.Z. prepared the materials, conducted the experiments, and analyzed all data; J.L.B. and A.V. led on manuscript preparation, while all other authors revised the manuscript and approved the final version.

## Supplementary Material

giab013_GIGA-D-20-00197_Original_Submission

giab013_GIGA-D-20-00197_Revision_1

giab013_Response_to_Reviewer_Comments_Original_Submission

giab013_Reviewer_1_Report_Original_Submission Xupo Ding -- 8/24/2020 Reviewed

giab013_Reviewer_1_Report_Revision_1 Xupo Ding -- 12/28/2020 Reviewed

giab013_Reviewer_2_Report_Original_Submission Fanna Maina -- 9/3/2020 Reviewed

giab013_Reviewer_2_Report_Revision_1 Fanna Maina -- 12/31/2020 Reviewed

giab013_Reviewer_3_Report_Original_Submission Gincy P. Thottathil -- 9/13/2020 Reviewed

giab013_Supplemental_Files
