# Supplementary material for: Genome sequence and genetic diversity analysis of an under-domesticated orphan crop, white fonio (Digitaria exilis)
Source: Gigascience. 2021 Mar 12;10(3):giab013. doi: 10.1093/gigascience/giab013 (PMC7953496; doi:10.1093/gigascience/giab013)
Supplement: giab013_GIGA-D-20-00197_Revision_1 [file giab013_giga-d-20-00197_revision_1.pdf]

GigaScience

Genome sequence and genetic diversity analysis of an under-domesticated orphan crop, white fonio (Digitaria exilis)

--Manuscript Draft--

Manuscript Number:	GIGA-D-20-00197R1
Full Title:	Genome sequence and genetic diversity analysis of an under-domesticated orphan crop, white fonio (Digitaria exilis)
Article Type:	Data Note
Funding Information:	Giles Fellowship from the University of Georgia — Dr Jeffrey L. Bennetzen<br>H2020 European Research Council (833522) — Dr Yves Van de Peer<br>Seed Biotechnology Center, UC Davis — DR. Allen Van Deynze<br>ICRISAT — Dr Jason Wallace<br>McKnight Foundation — Dr Moussa D. Sanogo
Abstract:	Background: Digitaria exilis , white fonio, is a minor but vital crop of West Africa that is valued for its resilience in hot, dry and low fertility environments and for the exceptional quality of its grain for human nutrition. The crop is plagued, however, by a low degree of improvement.<br>Findings: We sequenced the fonio genome with long-read SMRT-cell technology, yielding a ~761 Mb assembly in 3333 contigs (N50 1.73 Mb, L50 126). The assembly approaches a high level of completion, with a BUSCO score of greater than 99%. The fonio genome was found to be a tetraploid, with most of the genome retained as homoeologous duplications that differ overall by ~4.3%, neglecting indels. The two genomes within fonio were found to have begun their independent divergence ~3.1 million years ago. The repeat content (>49%) is fairly standard for a grass genome of this size, but the ratio of Gypsy to Copia LTR-retrotransposons (~6.7) was found to be exceptionally high. Several genes related to future improvement of the crop were identified including shattering, plant height and grain size. Analysis of fonio population genetics, primarily in Mali, indicated that the crop has extensive genetic diversity that is largely partitioned across a north-south gradient coinciding with the Sahel and Sudan grassland domains.<br><br>Conclusions: We provide a high-quality assembly, annotation and diversity analysis for a vital African crop. The availability of this information should empower future research into further domestication and improvement of fonio.
Corresponding Author:	Allen Van Deynze, Ph.D<br>University of California Davis<br>Davis, CA UNITED STATES
Corresponding Author Secondary Information:	
Corresponding Author's Institution:	University of California Davis
Corresponding Author's Secondary Institution:	
First Author:	Jeffrey L. Bennetzen
First Author Secondary Information:	
Order of Authors:	Jeffrey L. Bennetzen<br>Shiyu Chen<br>Xiao Ma<br>Xuewen Wang

Powered by Editorial Manager® and ProduXion Manager® from Aries Systems Corporation

	Anna E. J. Yssel
	Srinivasa R. Chaluvadi
	Matthew Johnson
	Prakash Gangashetty
	Falalou Hamidou
	Moussa D. Sanogo
	Arthur Zwaenepoel
	Jason Wallace
	Yves Van de Peer
	Allen Van Deynze, Ph.D
**Order of Authors Secondary Information:**	
**Response to Reviewers:**	See enclosed file under Personal Cover-Response to Review and Manuscript R1 tracked changes.
**Additional Information:**	
**Question**	**Response**
Are you submitting this manuscript to a special series or article collection?	No
**Experimental design and statistics**<br><br>Full details of the experimental design and statistical methods used should be given in the Methods section, as detailed in our Minimum Standards Reporting Checklist. Information essential to interpreting the data presented should be made available in the figure legends.<br><br>Have you included all the information requested in your manuscript?	Yes
**Resources**<br><br>A description of all resources used, including antibodies, cell lines, animals and software tools, with enough information to allow them to be uniquely identified, should be included in the Methods section. Authors are strongly encouraged to cite Research Resource Identifiers (RRIDs) for antibodies, model organisms and tools, where possible.<br><br>Have you included the information	Yes

requested as detailed in our Minimum Standards Reporting Checklist?	
**Availability of data and materials**<br><br>All datasets and code on which the conclusions of the paper rely must be either included in your submission or deposited in publicly available repositories (where available and ethically appropriate), referencing such data using a unique identifier in the references and in the “Availability of Data and Materials” section of your manuscript.<br><br>Have you have met the above requirement as detailed in our Minimum Standards Reporting Checklist?	Yes

Powered by Editorial Manager® and ProduXion Manager® from Aries Systems Corporation

Manuscript

Click here to access/download;Manuscript;FonioMs16 clean (1).docx

Genome sequence and genetic diversity analysis of an under-domesticated orphan crop, white fonio (*Digitaria exilis*)

Xuewen Wang[1,]*, Shiyu Chen[2,]*, Xiao Ma[3,]*, Anna E. J. Yssel[4,5,], Srinivasa R. Chaluvadi[1], Matthew S. Johnson[6], Prakash Gangashetty[7], Falalou Hamidou[7], Moussa D. Sanogo[8], Arthur Zwaenepoel[3], Jason Wallace[9], Yves Van de Peer[3,4,10] Jeffrey L. Bennetzen[1,], and Allen Van Deynze[2#.]

[1]Department of Genetics, University of Georgia, Athens, GA 30602 USA,

[2]Department of Plant Sciences, Seed Biotechnology Center, University of California, Davis, CA 95616 USA,

[3]Department of Plant Biotechnology and Bioinformatics, Ghent University, Ghent, Belgium VIB - UGent Center for Plant Systems Biology, Technologiepark 71, Ghent, Belgium,

[4]Centre for Microbial Ecology and Genomics, Department of Biochemistry, Genetics and Microbiology, University of Pretoria, Pretoria 0028, South Africa,

[5]Centre for Bioinformatics and Computational Biology, Department of Biochemistry, Genetics and Microbiology, University of Pretoria, Pretoria 0028, South Africa,

[6]Institute of Plant Breeding, Genetics, and Genomics, University of Georgia, Athens, GA 30602 USA,

[7]International Crops Research Institute for the Semi-Arid Tropics (ICRISAT), BP 12404, Niamey, Niger,

[8]Institut d'Economie Rurale, Ministere de l'Agriculture, Cinzana, BP 214, Ségou, Mali

[9]Department of Crop and Soil Sciences, University of Georgia, Athens, GA 30602 USA,

[10]College of Horticulture, Nanjing Agricultural University, Nanjing, China.

*These authors contributed equally to this work.

[#]corresponding author. avandeynze@ucdavis.edu. Ph: +1 (530) 304-9329

ORCIDs:

Name	ORCID ID
Xuewen Wang	0000-0003-2820-9255
Shiyu Chen	0000-0003-0102-8112
Xiao Ma	0000-0003-4787-9318
Anna. E. J. Yssel	0000-0003-1165-3237
Srinivasa Chaluvadi	0000-0003-2865-4156
Matthew S. Johnson	0000-0002-1786-103X
Prakash Gangashetty	0000-0002-6766-1415
Falalou Hamidou	0000-0002-7171-1497
Moussa D. Sanogo	0000-0003-4617-1756
Arthur Zwaenepoel	0000-0003-1085-2912
Jason Wallace	0000-0002-8937-6543
Yves Van De Peer	0000-0003-4327-3730
Jeffrey L. Bennetzen	0000-0003-1762-8307
Allen Van Deynze	0000-0002-2093-0577

Abstract

Background: *Digitaria exilis*, white fonio, is a minor but vital crop of West Africa that is valued for its resilience in hot, dry and low fertility environments and for the exceptional quality of its grain for human nutrition. The crop is plagued, however, by a low degree of plant breeding and improvement.

Findings: We sequenced the fonio genome with long-read SMRT-cell technology, yielding a ~761 Mb assembly in 3329 contigs (N50 1.73 Mb, L50 126). The assembly approaches a high level of completion, with a BUSCO score of greater than 99%. The fonio genome was found to be a tetraploid, with most of the genome retained as homoeologous duplications that differ overall by ~4.3%, neglecting indels. The two genomes within fonio were found to have begun their independent divergence ~3.1 million years ago. The repeat content (>49%) is fairly standard for a grass genome of this size, but the ratio of *Gypsy* to *Copia* LTR-retrotransposons (~6.7) was found to be exceptionally high. Several genes related to future improvement of the crop were identified including shattering, plant height and grain size. Analysis of fonio population genetics, primarily in Mali, indicated that the crop has extensive genetic diversity that is largely partitioned across a north-south gradient coinciding with the Sahel and Sudan grassland domains.

Conclusions: We provide a high-quality assembly, annotation and diversity analysis for a vital

African crop. The availability of this information should empower future research into further domestication and improvement of fonio.

Key Words: domestication, gene amplification, gene loss, millet, polyploidy

Data Description

Background information

White fonio (*Digitaria exilis*, NCBI:txid1010633) is a vital cereal crop of West Africa, where it is commonly known as fonio or acha. A related *Digitaria* species, black fonio (*D. iburura*), is a very minor crop, mostly of Nigeria, Benin and Togo. Fonio (*D. exilis*) has an exceptionally small but very nutritious grain, with both high protein and high dietary fiber content [1-3]. Fonio can mature in as little as eight weeks after planting, and is commonly grown without fertilizer or irrigation on poor quality soils in dry regions of the Sudan grasslands and Sahel. Although yields are low, the West African crop is harvested in early summer, where it fills a vital dietary gap before the maturation of sorghum or pearl millet crops in the same region. Perhaps no other crop deserves the title "orphan" more, because research attention on fonio has been minimal [4].

Wild *D. exilis* (sometimes called "hungry rice") and other West African *Digitaria* have been harvested by farmers in times of famine throughout recorded history[4], but very little improvement has been made to the domesticated crop, at least partly evidenced by the fact that no controlled cross between fonio varieties has been substantiated. Fonio was probably domesticated in West Africa, presumably before the arrival of pearl millet or sorghum from Central and East Africa [5], as is suggested by the importance of fonio in Dogon and other creation myths [4]. Applying the term "domesticated" to fonio cultivars is, however, something of a stretch. Fonio cultivars do not exhibit the full set of domestication traits, in that they exhibit the shattering (grain release at maturity) and day-length dependence traits that have been selected against by early farmers across virtually all cereal crops [6, 7]. The selected mutations to non-shattering and daylength independence are routinely recessive, so the absence of these agricultural improvements may be an outcome of the polyploid nature of the fonio genome [8].

As an orphan crop, fonio has received very little research attention. Over the last 20 years, for instance, only nine refereed publications report any new investigation of any aspect of fonio biology, although an additional 30 plus publications in that time period investigated fonio agronomy, cultural significance or nutritional properties [9, 10]. In 2007, Adoukonou-Sagbadja and colleagues [11] published a DNA marker-based analysis of fonio genetic diversity, and there is some transcript sequence data at NCBI [12]. Beyond this, most fonio investigations have been

conducted in West Africa to determine appropriate conditions for subsistence farmers to grow and/or process the grain from local landraces. In contrast, several other orphan cereal crops of Africa and Asia have begun to receive extensive attention, including comprehensive analyses of germplasm resources, even to the extent of full genome sequence analysis. Three of these cereals with relatively deep recent analyses are, like fonio, panicoid grasses: foxtail millet (*Setaria italica*), pearl millet (*Cenchrus americanus*) and proso millet (*Panicum miliaceum*) [13-15]. With these panicoid grass resources, and a comparative genomics strategy [16], it should be possible to rapidly elevate fonio research to benefit fonio consumers and producers. This manuscript describes our genomic sequence analysis of the fonio landrace Niatia, and a genetic comparison of fonio germplasms from across West Africa.

Plant material and nucleic acid preparation

Fonio millet (cv. Niatia) seed were obtained from Dr. Sara Patterson (University of Wisconsin, USA) which was collected in Mali at GPS coordinates 3.9861 W, 17.5739 N. Niatia is a popular local variety in Mali [17] (see Genetic Diversity for Nagoya protocol and germplasm access). The seed were multiplied in a University of Georgia greenhouse. Seeds collected from a single plant were used for all DNA isolation. The seeds were surface sterilized with 8% sodium hypochlorite (Bioworld, United States) for 10 min, followed by three rinses with sterile distilled water. The plants were grown in standard potting soil (Fafard® 4M Sungro Professional Growing Mix (Sungro Horticulture, USA) in a greenhouse (with 14 h daylight and day/night temperatures of 26/20˚C). They were watered daily to ~70% soil water-holding capacity. The leaves of four-week-old plants were used for DNA isolation, using a previously described protocol [18]. Briefly, leaf tissue (2.5g) was ground in liquid nitrogen. After lysing in 15 ml of 2X extraction buffer (100 mM Tris–HCl pH 8.0, 1.4 M NaCl, 20 mM EDTA, 2% w/v CTAB with 10 µl/ml β-mercaptoethanol) and extracted with chloroform/isoamyl alcohol twice, the aqueous phase was then transferred to 3 to 3.5 volumes of precipitation buffer (50 mM Tris–HCl pH 8.0, 10 mM EDTA, 1% w/v CTAB). The sample was incubated overnight at room temperature to precipitate the DNA. After centrifugation at 3500 rpm for 15 min., the DNA pellet was washed with ddH_2O and centrifuged for 10 min. Then, 5 ml of 1.5 M NaCl and 6 µl of 10 mg/ml RNaseA was added to the pellet and incubated at 37°C until completely re-suspended. A chloroform extraction was performed as above to remove RNaseA and any additional

contaminants. The aqueous phase was collected and DNA was precipitated and washed with ethanol. The pellet was then re-suspended in 100 µl ddH_2O.

PacBio SMRT sequencing, sequence polishing and genome assembly

DNA samples were used to construct a PacBio (Pacific Biosciences, Menlo Park, USA) SMRT sequencing library according to manufacturer recommendations at the University of California at Davis Genome Center. Fragments >10 kb were selected for sequencing via BluePippen (Sage Science, LLC, Beverly, USA). A total of 88 Gb of raw PacBio reads from 76 SMRT cells were passed through the secondary analysis pipeline in SMRT Link (v6.0, [19] and filtered for read quality higher than 0.75 and length longer than 1 kb. The resultant 75 Gb of filtered reads were assembled in Canu (v1.8, RRID:SCR_015880, [20]) with the default settings for raw PacBio reads.

Racon (Racon, RRID:SCR_017642) was used to polish the original assembly for two rounds with the Canu-corrected PacBio reads. Sequentially, Arrow (VariantCaller v2.3.3) and Pilon (v1.23, RRID:SCR_014731) were used to further polish the assembly with 36 Gb of Illumina paired-end reads obtained on the HiSseq4000 (RRID:SCR_016386) at the Georgia Genomics and Bioinformatics Core at the University of Georgia.

The final assembly (Niatia v1.0) has a total length of 760.66 Mb and 3329 contigs, with N50 of 1.73 Mb (L50 of 126) and N90 of 75.85 kb (L90 of 889). The longest contig is 10.17 Mb and the shortest contig is 1013 bp with a mean of 228.5 kb. We compare the quality of the our genome with that of CM05836 [21] which was assembled using short-reads, linked reads and Hi-C. Although scaffold size is larger for the aforementioned genome, our genome has much better contiguity than CM05836 [21] as seen by N50 (1,734 kb vs 78 kb) and L50 (8 vs 2,624). (Suppl **Table 1**). Scaffolding is expected to be higher in the latter genome as Hi-C technology was used that associates contigs on the same histone protein regardless of their size, but the Niatia genome shows much greater contiguity. In order to see the high contiguity in our genome assembly in detail, we took two of our medium sized contigs (tig00001331 and tig00010942) as examples showing a dramatic improvement in contiguity in our genome, emphasizing the importance of long reads on assembly and annotation (see Annotation below). This is further exemplified by comparing two random medium sized contigs, tig00001331 corresponding to 100 consecutive

segments anchored on the same chromosome 3B and tig00010942 corresponding to 65 consecutive segments on the chromosome 5A of the CM05836 [21] genome (**Suppl Fig. 1**).

Estimation of genome size and heterozygosity

Kmer Analysis Toolkits [22] was used to count kmers in Illumina raw reads and to compare the results with the kmers counted from the genome assembly at several different kmer sizes, from 17-30. These all yielded similar results, but with a somewhat larger fonio genome predicted at smaller kmer lengths. The distribution of kmer counts was modeled and the heterozygosity level was estimated using GenomeScope2.0 [23].

Two distinct peaks were observed in the raw read kmer distribution. We interpret the peaks at ~50 and ~100 counts/coverage as the two subgenomes in fonio (**Suppl. Fig. S2**). Genome size estimated from the peaks was 668-707 Mb, depending on the kmer size employed. This range of values is low compared to previous results from flow cytometry that indicated a genome size range of 830-1000 Mb for a broad selection of *D. exilis* germplasm [4]. The underestimate is likely due to polyploidy confounding duplicated genes both within and among subgenomes. Single nucleotide variation was estimated to be 4.3% when comparing the A and B genomes in this tetraploid, but slightly less than 0.01% heterozygosity was observed within either the A or B genomes, as assayed by kmer allelic ratios. Kmer counts in the assembled genome suggests that the peak at 100 counts represents common sequences between the two subgenomes, and the kmers under the peak at 50 counts represent the divergent regions between the two subgenomes.

Repeat annotation

Repeated sequences were mined and annotated with a combination of *de novo* and homology-based methods. First, simple sequence repeats were identified and masked with GMATA [24]. Long-Terminal-Repeat-Retrotransposons (LTR-RTs) were identified *de novo* using the bioinformatic tools LTR_FINDER (LTR_Finder, RRID:SCR_015247) [25] and LTRharvest (LTRharvest, RRID:SCR_018970) [26] that employ structural criteria to find intact LTR-RTs, followed by LTR_retriever analysis [27] to minimize false positives. SINE scan (version 1.1.1) [28] was used to find small interspersed nuclear elements (SINEs), a class of retroelements, and

these were confirmed by manual investigation. Long interspersed nuclear elements (LINEs), another class of retroelements, were found with MGEscan-nonLTR (version 2) [29]. Small DNA transposable elements (TEs) were found with MITE Tracker [30] and HelitronScanner [31] was used to identify the DNA transposons called *Helitrons*. All of the TEs from the genome assembly were used to generate a fonio-specific TE library, with individual TE families named according to the prevalent current nomenclature system [32]. The fonio TE library was compared to the multispecies repeat repository called Repbase [33] to validate annotations, and to discover any additional candidate repeats represented in Repbase. Then, the fonio TE library was used to identify both full-length and truncated TE elements by homologous search with RepeatMasker version 4.0.7 (RepeatMasker, RRID:SCR_012954) [34] in the genome assembly. Parameter settings were adopted from the analysis described in a previous publication [35]. The predicted insertion dates of intact LTR-RTs were calculated with LTR_retriever (LTR_retriever, RRID:SCR_017623) [27]. The SSRs and TEs were masked by Ns and a TE annotation file in GFF3 format was generated for subsequent gene annotation. Types and abundances of TEs and other repeats discovered in the fonio genome are presented in **Table 1**.

Table 1. Summary of repeat sequence properties in the genome assembly.

Class	Subclass	Type	Number of families	Number of repeats	Length (Mb)	Percent of genome
Class I TEs						
Retroelements	LTR-RT	*Copia*	353	45,194	22.8	3.0
		Gypsy	1223	125,773	153.9	20.2
		Other	824	90,110	57.8	7.6
	LINE	I	17	3040	1.5	0.2
	SINE		3790	181,505	30.6	4.0
Class II TEs						
DNA Transposons	TIR	CACTA	348	42,737	7.4	1
		Mutator	34	8493	1.8	0.2
		PIF	120	13,973	2.4	0.3
		Tc1	896	124,252	21.5	2.8
		hAT	93	13097	2.5	0.3
	Helitron	*Helitron*	313	104,271	21.6	2.8
Tandem repeats	SSRs			133,570	5.9	0.8
Unclassified repeats	(Repbase)				48	6.3
	Total				329.8	49.7

Transcriptome assembly, candidate gene annotation and BUSCO quality assessment

Illumina RNA sequencing data (paired-end 100 bp) of *Digitaria exilis* were downloaded from the NCBI Sequence Read Archive (accession number SRX1967865 [12]) from RNA consisting of ~80% inflorescence and ~20% leaf tissue. FastQC (FastQC, RRID:SCR_014583) [36] was used to evaluate data quality, and low-quality reads and adapter sequences were removed using Trimmomatic (Trimmomatic, RRID:SCR_011848) [37]. The remaining reads were aligned to the genome assembly using HISAT2 (HISAT2, RRID:SCR_015530) [38]. The spliced alignments were used as input for StringTie [39] and assembled into transcripts. TransDecoder, a companion software of the Trinity platform [40], was used to predict open reading frames.

For gene prediction and genome annotation, we used the Maker-P pipeline [41], in combination with Augustus (Augustus, RRID:SCR_008417) [42], SNAP [43] and GeneMark (GeneMark, RRID:SCR_011930) [44]. Augustus gene models came from the BUSCO (BUSCO, RRID:SCR_015008) [45] data set identified during the assembly (see below). GeneMark_ES was used to produce *ab initio* gene predictions. Detailed settings for each round of Maker can be found in the Supplemental Methods. The first round of gene prediction with Maker used the following inputs: the RNAseq assembly described in the previous section, protein fasta sequences from *S. bicolor* and *S. italica* [46] as well as the repeat models for *Digitaria* (described above), and the soft-masked genome assembly. A second round of Maker used as input the genome file, the annotation produced by the previous round and a SNAP species parameter/hmm file based on the prior annotation. Finally, the third round of Maker was run using the following input: the genome assembly, the annotation produced by round two and the GeneMark models. Functional annotation was done using the accessory scripts of Maker as described by Campbell and coworker [47]. Briefly, a BLAST [48] search against the Swissprot database was used to assign putative functions to the newly annotated gene models, while InterProScan 5 (InterProScan, RRID:SCR_005829) [49] was used to obtain domain information.

Following mapping of RNAseq data with HISAT2, 88% of the RNAseq reads could be well-aligned to the genome. Transcripts were assembled with Stringtie and ORFs were predicted with TransDecoder (TransDecoder, RRID:SCR_017647). A total of 58,305 candidate transcripts were obtained, of which 50,389 had predicted open reading frames.

Our first round of Maker predicted 60,300 protein-coding genes (based only on RNA

evidence and protein evidence from sorghum and Setaria). After the 2nd and 3rd round, where Augustus, SNAP and Genemark-ES models were included, the number of predicted protein coding genes increased to 67,921 and finally to 68,302. We removed 447 candidate genes that were judged as spurious because they were fragments of otherwise fully assembled genes in the annotation, so the final number of genes annotated as protein coding genes is 67,855. The statistics for the gene annotation can be found in the Supplemental Materials (**Table S2**). In total, 88.3% of the gene models were supported by RNAseq data. The Annotation Edit Distance (AED) measurements indicate how well an annotation agrees with overlapping evidence (protein, mRNA or EST data). In the fonio assembly, >90% of the gene models have an AED score less than 0.4%, indicating that gene models are well supported by evidence. The number of genes and gene model lengths are greater than that reported by Abrouk et al [21] for CM05836 (59,844) indicating the importance of long read assemblies and contiguity in genome assembly and annotation.

BUSCO v 4.0.2 [45, 50] analysis of the filtered predicted protein sequences against the reference set for plants, on the gVolante platform [51], showed that 98.1% of the BUSCO genes were found as complete genes, while this representation number increased to 99.3% if partially covered BUSCO genes were added compared to the 97.2 reported by Abrouk et al.[21]. A total of 11.6% of the BUSCO genes were single copy, while 86.5% of the BUSCO genes were found in duplicate. Approximately 1.2% of the BUSCO genes were fragmented and ~0.7% were missing.

A total of 4,741 non-coding RNAs (see **Suppl. Table S3**) were predicted with Infernal [52] by comparing the genome fasta file with the RFAM CM database, version 14.2 [53] using the protocol described in Kalvaru et al. [54]. Most of these non-coding RNAs were found to be tRNAs (31.2%), 5S rRNAs (12.2%) and snoRNAs (23.4%), as seen in other plant genomes.

Phylogenetic divergence and dating the most recent whole genome duplication

The coding DNA sequences (CDS) and annotations for *S. bicolor* and *S. italica* were downloaded from the PLAZA database [46]. K_S distribution analyses were performed using the

wgd package (v1.1) [47]. For each species, the paranome (entire collection of duplicated genes) was obtained with 'wgd mcl' using all-against-all BlastP [47] and MCL clustering [55]. K_S distributions were then constructed using 'wgd ksd' with default settings (using MAFFT for multiple sequence alignment [56], codeml for maximum likelihood estimation of pairwise synonymous distances [57], and FastTree (FastTree, RRID:SCR_015501) [58] for inferring phylogenetic trees used in the node weighting procedure. Anchors or anchor pairs (duplicates lying in collinear or syntenic regions of the genome) were obtained using i-ADHoRe [59] employing the default settings in 'wgd syn'.

We obtained gene families for a set of nine species in the Poaceae family using OrthoFinder (OrthoFinder, RRID:SCR_017118) with default settings [60]. All sequence data were obtained from PLAZA [46]. From this set of gene families, we identified all gene families that were single-copy in all species but duplicated in *D. exilis*, and where the *D. exilis* duplicates were anchor pairs (1,967 gene families). For these gene families, we performed pre-alignment homology filtering using PREQUAL [61] and multiple sequence alignment of the masked amino acid sequences using MAFFT (MAFFT, RRID:SCR_011811) [56]. For each multiple sequence alignment, we obtained the corresponding codon-level nucleotide alignment. For each obtained nucleotide alignment, we sampled tree topologies from the posterior using MrBayes v3.2 (MrBayes, RRID:SCR_012067) [62] under the GTR model with a discrete Gamma mixture for relative substitution rates across sites (using four classes), sampling every 10 iterations, for a total of 250,000 iterations. We then identified all gene families for which the expected species tree topology had posterior probability above 0.9, resulting in a set of 1,242 gene families. A concatenated codon alignment was obtained for these families, which was in three partitions corresponding to each codon position. We then performed posterior inference of substitution rates and divergence times for the partitioned alignment using MCMCTree [55, 63] using the multivariate Normal (MVN) approximation of the likelihood (where the MVN approximation was based on the maximum likelihood estimates under the GTR model with Gamma distributed relative rates across sites (5 categories). We used a Gamma (2, 11) prior for the mean substitution rate per site per 100 My (million years), based on a rough estimate of the substitution rate under the molecular clock with a root age of 50 My obtained using baseml from the PAML package [53]. We use an independent log-normal rates relaxed molecular clock prior on branch-specific substitution rates, using a Gamma (2, 10) prior for the variance parameter of

the clock. We set the birth-death-sampling prior such that a uniform prior over node ages is obtained. We include two fossil calibrations. First, we used a minimum age for the *Oryza* - *Hordeum* divergence of 34 My based on the review of Iles et al. [64]. Next, a secondary calibration for the root based on previous dating studies included in the Time Tree [65] database was used, where we excluded all time estimates younger than the 34 My constraint and older than 80 My. We then fitted a log-normal distribution to the age estimates in the time tree data, which we approximated by a Gamma (47,100) distribution. We used MCMCTree to obtain 5000 from the posterior sampling every 200 iterations after a burn-in of 50,000 iterations. We compared two independent runs with each other to verify convergence and with a run of the MCMC algorithm under the prior alone to compare the posterior distribution for the node ages to the effective prior implied by the fossil calibrations (**Suppl. Fig. S3**). The results of this analysis provide the phylogenetic tree shown in **Figure 1D**.

Transposable element properties

The ~42.6% TE content of the fonio genome is a minimal estimate, given that degraded TE fragments are often missed by the *de novo* discovery analysis that was employed. This underestimation is routine in other plant genome annotations as well [66], so it is reasonable to compare TE descriptions across plant genomes. In fonio, the very high level of *Gypsy* LTR-RTs compared to *Copia* LTR-RTs is exceptional. Although most grass genomes have more *Gypsy* TEs than *Copia* (for instance, ~50% *Gypsy* and ~25% *Copia* in the ~2.4 Gb maize genome [67] or ~36% *Gypsy* and ~33% *Copia* in the ~2.8 Gb pearl millet genome [14], the ~6.7:1 *Gypsy* to *Copia* ratio in the ~900 Mb fonio genome is unprecedented. One should remember, however, that the diploid constituent genomes of fonio are ~450 Mb, so somewhat similar results are observed in other small panicoid genomes like sorghum (~750 Mb) and rice (~430 Mb), with *Gypsy*/*Copia* of ~3.7 and ~4.9, respectively [68]. This fonio observation is surprising because the quantity of *Gypsy* LTR-retrotransposons is the major determinant of genome size in grasses [69], so one would expect higher *Gypsy* to *Copia* ratios as genome size increases, rather than the opposite that we observe. These results suggest that either different factors initiate *Gypsy* amplification bursts than *Copia* amplifications, or that *Copia* elements are particularly sensitive to shared activation factors. It would be useful to investigate additional *Digitaria* species to see if

this *Gypsy*/*Copia* ratio trait is shared by other close relatives, and thus a possible outcome of common ancestral properties.

Analysis of LTR-RT insertion dates demonstrated that most of the elements inserted within the last 2 My. This high level of recent activity is a standard observation in the grasses, at least partly caused by the fact that the rapid DNA removal by accumulated small deletions quickly excise and otherwise obscures any DNA that is not under positive selection [70, 71].

Whole genome duplication and subsequent stability

We inferred whole-paranome and one-vs.-one ortholog K_S distributions and performed syntenic analyses to further assess the clear signature of a relatively recent whole-genome duplication (WGD) in *Digitaria exilis.* K_S distributions present a very clear signature of WGD in the recent evolutionary past of *D. exilis,* with this event not shared with the closest relative in our analyses (*S. italica)* **(Figure 1A)**. We note that a trace of an older, likely Poaceae-shared WGD [72] event was also clearly observed in both the whole-paranome and anchor pair K_S distributions of *D. exilis,* coinciding with similar signatures in sorghum and Setaria (**Figure 1B**). Analysis of co-linearity and synteny show that the genome of *D. exilis* is still largely conserved in duplicate (**Figure 1C**). Phylogenetic divergence time estimation (**Figure 1D**) estimated the timing of the WGD event (or divergence of parental genomes in the case of an allopolyploidy event) at ~3.1 million years ago (mya) with a 95% posterior uncertainty interval of (2.2, 4.2 My) and the divergence of *Digitaria* from *Setaria* at 17.8 (12.5, 23.1) mya; with these estimates associated with a posterior mean substitution rate across the three codon positions of 2.5×10^{-9} (1.1×10^{-9}, 5.0×10^{-9}) substitutions per year per site. This is consistent with CM05836 [21]. The closest relative to fonio with a whole genome sequences would be *Panicum miliaceum, S. italica* and *C. americanus*. The diploid ancestor to *D. exilis* is not clear [73].

It is interesting that **Figure 1C** shows extreme conservation of gene content and order across long scaffolds, but also the presence of large rearrangements that differentiate chromosome-size blocks. This suggests a possible selection for major rearrangements after the polyploids were formed, perhaps to minimize tetrasomic inheritance [74, 75].

In the ~3.1 My since the latest WGD, most of the duplicated genes have had both copies retained. For instance, the BUSCO gene set yielded 86.5% of the genes still in a duplicated state.

Our genome assemblies did not yield complete chromosomes, so we could not investigate the details of major chromosomal rearrangements, preferential gene loss (also known as fractionation), or parent-specific gene expression differences that might differentiate the two ancestral genomes in this tetraploid [76]. The large stretches of gene content and gene collinearity retention observed between our largest contiguous assemblies (**Figure 1C**) do demonstrate, however, that there has been no large number of small rearrangements of these genomes over the last 3.1 My.

Expansions and contraction of gene families

In order to see the expansions and contractions of gene families, broomcorn millet (*Panicum miliaceum* L.) was added in the phylogenetic analysis, as it experienced a recent tetraploidization estimated at ~5.8 MYA that is similar to fonio.

Based on sequence homology, we assigned 58,459 genes to 20,003 families, 14,549 of which have expanded in the fonio genome. Expansion in a similar number of gene families (11,819) was also observed in the broomcorn millet genome, also an allotetraploid crop. Of the fonio gene families, 57.4% contain two copies (the most abundant category in these ten species) and 30.4% contain more than two copies (**Figure 2**). Most (~90%) of the two-copy gene families of fonio are located in syntenic blocks, indicating that the expansion was mainly due to the recent WGD event (**Figure 2** and **Suppl. Fig. S4**).

In addition to the majority of multi-copies genes, there are many (~12.1% of the total) that are single-copy genes, and thus a likely outcome of at least some deletion after polyploidy. GO enrichment analyses of contracted genes (1 copy **Suppl. Fig. S5**) and expanded genes (>2 copies, **Suppl Fig. S6**) relative to *O. sativa* were performed. The analyses identifies negative regulators and recognition factors for biotic and abiotic stresses, as well pollen/fertility recognition as single copy genes. In contrast, there is general expansion of gene families encoding positive regulators of multiple copy genes. These results suggest that further analysis of these genes may reveal their roles in heat and drought stress tolerance, and in understanding of crossing barriers in fonio.

Candidate domestication genes

Improvement of fonio will require further domestication, particularly to solve the issues of shattering and lodging. This process should be greatly assisted by the provision of a comprehensive genome sequence.

In rice, sorghum and maize, mutations in the gene SSH1 (SUPPRESSION of SEED SHATTERING-1) are associated with panicle retention of the grain after seed maturation (the "non-shattering" trait) in domesticated accessions [77]. Nine sequenced grass genomes were scanned with OrthoFinder (as described in the section "Phylogenetic divergence and dating the most recent whole genome duplication") to find the orthologues of this gene. The gene family fasta files were used to construct trees using Mafft and Iqtree, trees were visualized in FigTree. Interproscan was used to annotate the proteins with their pFam domains, and alignments were visualized in Geneious Prime [78].

Fonio has 4 genes related to SSH1, but the phylogenetic tree indicated that two are more closely related to the rice SSH1 gene associated with shattering than to the other SSH1-like gene in rice (**Suppl. Fig. S7**). Other species included in our dataset have between 1 and 3 SSH1-like genes (**Suppl. Table S4**). The extra copies in *D. exilis* are expected because of its polyploid nature, and thus can explain why no ancient or modern farmers have detected recessive single gene mutations at each of these loci in a single fonio plant. By modern forward or reverse genetic and breeding techniques, inactivation and selection of both of these genes should be targeted in order to solve the shattering problem in fonio.

Inactivation of the *dw3* (Dwarfing-3) genes of sorghum is responsible for the semi-dwarf trait that diminishes lodging and thereby greatly improves yield and input response in this important crop of arid and semi-arid agriculture [79]. Inactivation-mutant orthologues of the same gene are also responsible for the pearl millet cultivars with highest lodging resistance and the highest grain yield [80]. Hence, orthologues of *dw3* also should be targets for inactivation-mutation and molecular breeding in fonio. Once again, fonio has more copies of this gene than do any of the other grasses screened, all of which are diploids (**Suppl. Fig. S8 and Table S5**).

The GW2 (GRAIN WEIGHT-2) gene controls seed weight in wheat and rice, with inactivation of the gene leading to larger grain [81, 82]. Orthofinder results indicated that members of this gene family are present in single copy in all of the examined grass species, except fonio and maize (**Suppl. Fig. S9 and Table S6**). The two copies in *D. exilis* only differ from each other by 3 amino acid residue substitutions. The fonio genes were found to be nearly

identical to the unmutated GW2 version that yields smaller grain in rice and wheat (data not shown). Although increased seed weight does not always increase yield (due to correlated traits, like seed number), it is a particularly important trait in fonio to enable sowing for uniform stands and mechanical threshing.

Genetic diversity

Fonio genetic diversity was assessed using 184 samples from ~130 accessions collected from Mali and Niger, signatories to the Cartagena Protocol on Biosafety (**Suppl. Table S7**). Consistent with the Nagoya Protocol and the third objective of the Convention on Biological Diversity of access and benefit sharing, fonio materials from Mali were collected in Mali by Institut d'Economie Rural (IER) while those from Niger were collected in Niger by Institute National de Recherche Agronomique du Niger (INRAN) and conserved at the ICRISAT Niamey genebank. Authors Sanogo, Hamidou and Gangashetty were involved in the germplasm collection, seed conservation at the genebank and/or DNA extraction from young seedlings. All DNA samples or seed were sent to the USA for analysis for research purposes only. This research has no direct commercial application.

Seedlings of each sample were grown at the respective institutions in West Africa, and DNA was extracted from young leaves with a QIAGEN DNeasy Plant Mini Kit (Germantown, USA). Lyophilized DNA was then sent to Data2Bio (Ames, USA) for tunable genotyping-by-sequencing (tGBS) using 2-bp selection and 5 runs on an Ion Torrent Ion Proton Instrument (Thermo Fisher Scientific, Waltham, USA). The resulting raw sequences were quality-trimmed by Data2Bio, which removed bases with PHRED quality scores <15. These trimmed sequences were then aligned to the genome assembly with GSNAP v2020-04-08 [83] using default parameters. SNPs were called using the bcftools mpileup command v1.9 [84] with max-depth set to 1000 and minimum base quality set to 20. Raw SNPs were then filtered using TASSEL v5.2.40 [85], custom R scripts with R v3.5.1 [86], and bcftools to include only sites with ≤25% heterozygosity, ≤500 total read depth, ≤60% missing data, and ≥2.5% minor allele frequency (**Suppl. Table S8**). Population substructure was determined with fastStructure v1.0 [87], testing from 1 to 10 population clusters and identifying the optimal number with the included chooseK.py program. This identified 3 clear clusters of material, with genetic separation strongly correlated with geography (**Figure 3A**). The genetic distinctions among these clusters are clear

when plotting the genetic principal coordinates and relationship dendrogram (**Figure 3B**). A small number of accessions (<5) appear "misplaced" on the geographic map, which could be due to recent transfer of germplasm or human error during collection, storage, or processing. Geographic clustering can reflect both human trafficking of seed stocks and the genetic basis of local adaptation. Further (both broader and deeper) germplasm analyses will be useful for resolving these issues.

Conclusions

Genome analysis of any polyploid is challenging, especially when no diploid ancestors are known. Our sequence of the white fonio (*D. exilis*) genome indicates its recent tetraploid origin and the retention of most of the genes duplicated in this process. This retention of duplicated genes likely explains why recessive mutations for important agronomic traits like shattering, seed size, semi-dwarfism and others like day-length dependence have not yet been detected in fonio. However, it is now possible to identify such mutations by using modern mutation detection schemes, like those used for the tetraploid cereal *Eragrostis tef* [88]. One purpose for generating a fonio genome sequence was to attract molecular genetics researchers into the study of this crop, and thereby enable hypothesis-driven breeding through genomics-assisted selection. If future researchers develop a transformation technology for fonio [89] or develop other genome editing strategies [90], then directed mutagenesis could be used to knock out pairs of these domestication genes in a single step [91].

The importance of correcting such problems as shattering, seed size, lodging in fonio cannot be overestimated. Until shattering is solved, farmers will continue to be required to harvest before grains fully mature, thus dramatically decreasing overall yield. Without semi-dwarf varieties, already serious lodging problems in fonio will continue to prohibit the use of more inputs (because fertilizer increases plant height and thus lodging) or even the selection of larger grain yield from the panicles, because greater weight on the top of the plant can cause more lodging. The same will almost certainly be true for fonio, hence providing a partial explanation for its tiny seed size in cultivated landraces. With domestication traits fully penetrant into fonio cultivars, one can expect dramatic increases in fonio performance, with expectations of a two-fold or greater yield enhancement easily within the short-term range of possibilities.

The absence of an outcrossing protocol for fonio is another technical deficiency that severely limits this crop's potential for improvement. Our diversity analysis on cultivar Niatia indicates <0.01% heterozygosity, showing that crosses occur very rarely by natural processes. Hence, generating controlled crosses will probably require a serious dedication to this pursuit. Our results indicate a great deal of genetic variability within fonio landraces, so we have no doubt that hybridization could be used in breeding projects to optimize fonio germplasm quality for future W. African and other farmers.

Availability of Supporting Data

The genome and annotation can be accessed on the African Orphan Crops Consortium-specific branch of the ORCAE platform [92, 93] at: [94]. The GenBank project number for the assembly is PRJNA640067. All scripts for diversity analysis and data tables are available at [95] including full genotyping table. Genotyping table is also available at GenBank Project number PRJNA644458. All supporting data and materials are available at *GigaScience* GigaDB database [96].

Abbreviations

Dw3: dwarf3; Gb: gigabase; GW2: grain weight2; LINE: long interspersed nuclear element; LTR: long terminal repeat; LTR-RT: long terminal repeat retrotransposon; Mb: megabase; MITE: miniature inverted repeat transposable element; My: million years; mya: million years ago; NCBI: National Center for Biotechnology Information; ORF: open reading frame; SINE: small interspersed nuclear element; SMRT: single molecule, real time sequencing; SSH1: suppression of shattering1; SSR: simple sequence repeat; TE: transposable element; TIR: terminal inverted repeat transposable element.

Conflict of Interest

The authors declare that they have no competing interests.

Consent for publication

Not Applicable

Funding

JLB acknowledges the Giles Fellowship from the University of Georgia as a source of funding for this project. YVdP acknowledges funding from the European Research Council (ERC) under the European Union's Horizon 2020 research and innovation program (grant agreement No 833522). AV acknowledges funding from the Seed Biotechnology Center, University of California, USA. JGW acknowledges funding from the International Crops Research Institute for the Semi-Arid Tropics (ICRISAT) and the University of Georgia. MDS acknowledges funding from the McKnight foundation.

Author Contributions

J.L.B., J.W., Y.V., and A.V.D. conceived, designed and interpreted the study; S.C., X.M., X. W., A.E.J.Y., S.R.C., M.S.J., P.G., F.H., M.D.S., and A.Z. prepared the materials, conducted the experiments, and analyzed all data; J.L.B. and A.V. led on manuscript preparation, while all other authors revised the manuscript and approved the final version.

Acknowledgements:

Shu-Min Kao for providing scripts and Sara Patterson for providing Niatia seed and for helpful discussions. Oanh Nguyen from the UC Davis Genome and Biomedical Sciences Facility for technical expertise for sequencing with Pacific Biosciences. Armando Garcia-Llanos for DNA and library quality control for sequencing.

Figure Legends

Figure 1: Whole genome duplication and polyploidy analysis. (A) K_S estimation of age distribution for paralogs and orthologs of white fonio (*Digitaria*) and some close relatives. The distribution in light pink represents the entire white fonio paranome, while the distribution in darker pink represents the anchor points (duplicated genes lying in syntenic or collinear regions (see C)). Distributions in black, dark green and light green represent the one-vs.-one ortholog comparisons between *Digitaria-Setaria*, *Digitaria-Sorghum* and *Sorghum-Setaria*, respectively. (B) K_S distributions for paralogs of white fonio, sorghum and *Setaria* (zoom in), showing an older, likely Poaceae-shared, WGD. (C**)** Syntenic relationships between putative homoeologous contigs, with colored lines connecting homoeologous gene pairs in the white fonio genome assembly. (D) Time-calibrated phylogenetic tree of several major Poaceae lineages, including white fonio, based on 1242 gene families consisting of a single gene copy in each lineage and an anchor pair (A and B) in *Digitaria*. The time scale is shown in million years (My). See text for details.

Figure 2 The number of gene families that expanded or contracted during evolution mapped to the species phylogenetic tree in related Poaceae species.

Figure 3 – Fonio Genetic Diversity. The genetic diversity of fonio samples was surveyed by genotyping-by-sequencing. (A) Fonio samples originated from Mali and Niger. They separate into 3 primary subpopulations based on population structure analysis. Both principal coordinate analysis of the genetic diversity (B) and a neighbor-joining tree of the population (C) confirm these groupings. A few discrepancies between population assignment and geography may be due to recent long-distance germplasm exchanges or labelling errors during collection and storage.

References

1. Ballogou V, Soumanou M, Toukourou F and Hounhouigan J. Structure and nutritional composition of fonio (Digitaria exilis) grains: a review. International Research Journal of Biological Sciences. 2013;2 1:73-9.
2. Fanou N, Hulshof P, Koreissi Y and Brouwer I. NUTRITIVE VALUES OF FONIO AND FONIO PRODUCTS: P110-08. Annals of Nutrition and Metabolism. 2009;55.
3. Temple VJ and Bassa JD. Proximate chemical composition of Acha (Digitaria exilis) grain. J Sci Food Agr. 1991;56 4:561-3.
4. Vietmeyer N, Borlaugh N, Axtell J, Burton G, Harlan J and K R. Fonio (Acha). Lost Crops of Africa. Washington, DC: The National Academies Press; 1996. p. 59.

5. De Wet J. The three phases of cereal domestication. Grass evolution and domestication. 1992:176-98.
6. Aliero A and Morakinyo J. Photoperiodism in Digitaria exilis (Kipp) Stapf accessions. African journal of biotechnology. 2005;4 2:241-3.
7. Patterson SE, Bolivar-Medina JL, Falbel TG, Hedtcke JL, Nevarez-McBride D, Maule AF, et al. Are we on the right track: can our understanding of abscission in model systems promote or derail making improvements in less studied crops? Frontiers in plant science. 2016;6:1268.
8. Adoukonou-Sagbadja H, Schubert V, Dansi A, Jovtchev G, Meister A, Pistrick K, et al. Flow cytometric analysis reveals different nuclear DNA contents in cultivated Fonio (Digitaria spp.) and some wild relatives from West-Africa. Plant Syst Evol. 2007;267 1:163-76. doi:10.1007/s00606-007-0552-z.
9. Chukwurah PN, Uyoh EA, Usen IN, Ekerette EE and Ogbonna NC. Assessment of intra and inter species variation in antioxidant composition and activity in marginalized Fonio millet (Digitaria spp.). Journal of Cereals and Oilseeds. 2016;7 1:1-6.
10. Gigou J, Stilmant D, Diallo TA, Cisse N, Sanogo MD, Vaksmann M, et al. Fonio millet (Digitaria exilis) response to N, P and K fertilizers under varying climatic conditions in West Africa. Exp Agr. 2009;45 4:401-15.
11. Adoukonou-Sagbadja H, Wagner C, Dansi A, Ahlemeyer J, Daïnou O, Akpagana K, et al. Genetic diversity and population differentiation of traditional fonio millet (Digitaria spp.) landraces from different agro-ecological zones of West Africa. Theoretical and Applied Genetics. 2007;115 7:917-31.
12. Sarah G, Homa F, Pointet S, Contreras S, Sabot F, Nabholz B, et al. A large set of 26 new reference transcriptomes dedicated to comparative population genomics in crops and wild relatives. Mol Ecol Resour. 2017;17 3:565-80. doi:10.1111/1755-0998.12587.
13. Bennetzen JL, Schmutz J, Wang H, Percifield R, Hawkins J, Pontaroli AC, et al. Reference genome sequence of the model plant Setaria. Nature biotechnology. 2012;30 6:555-61.
14. Varshney RK, Shi C, Thudi M, Mariac C, Wallace J, Qi P, et al. Pearl millet genome sequence provides a resource to improve agronomic traits in arid environments. Nature biotechnology. 2017;35 10:969-76.
15. Zou C, Li L, Miki D, Li D, Tang Q, Xiao L, et al. The genome of broomcorn millet. Nature communications. 2019;10 1:1-11.
16. Bennetzen JL and Freeling M. The unified grass genome: synergy in synteny. Genome research. 1997;7 4:301-6.
17. Cruz J-F. Fonio. Upgrading quality and competitiveness of fonio for improved livelihoods in West Africa: Second activity report. 2008.
18. Murray M and Thompson WF. Rapid isolation of high molecular weight plant DNA. Nucleic acids research. 1980;8 19:4321-6.
19. Pacific Biosciences: Software Downloads. SMRT Link V 6.0. https://www.pacb.com/support/software-downloads (2020). Accessed June 21, 2020 2020.
20. Koren S, Walenz BP, Berlin K, Miller JR, Bergman NH and Phillippy AM. Canu: scalable and accurate long-read assembly via adaptive k-mer weighting and repeat separation. 2017; doi:10.1101/gr.215087.116.

21. Abrouk M, Ahmed HI, Cubry P, Šimoníková D, Cauet S, Pailles Y, et al. Fonio millet genome unlocks African orphan crop diversity for agriculture in a changing climate. Nature Communications. 2020;11 1 doi:10.1038/s41467-020-18329-4.
22. Mapleson D, Garcia Accinelli G, Kettleborough G, Wright J and Clavijo BJ. KAT: a K-mer analysis toolkit to quality control NGS datasets and genome assemblies. Bioinformatics (Oxford, England). 2017;33 4:574-6.
23. Ranallo-Benavidez TR, Jaron KS and Schatz MC. GenomeScope 2.0 and Smudgeplot for reference-free profiling of polyploid genomes. Nature Communications. 2020;11 1:1432. doi:10.1038/s41467-020-14998-3.
24. Wang X and Wang L. GMATA: an integrated software package for genome-scale SSR mining, marker development and viewing. Frontiers in plant science. 2016;7:1350.
25. Xu Z and Wang H. LTR_FINDER: an efficient tool for the prediction of full-length LTR retrotransposons. Nucleic acids research. 2007;35 suppl_2:W265-W8.
26. Ellinghaus D, Kurtz S and Willhoeft U. LTRharvest, an efficient and flexible software for de novo detection of LTR retrotransposons. BMC bioinformatics. 2008;9 1:18.
27. Ou S and Jiang N. LTR_retriever: a highly accurate and sensitive program for identification of long terminal repeat retrotransposons. Plant physiology. 2018;176 2:1410-22.
28. Mao H and Wang H. SINE_scan: an efficient tool to discover short interspersed nuclear elements (SINEs) in large-scale genomic datasets. Bioinformatics (Oxford, England). 2017;33 5:743-5.
29. Rho M and Tang H. MGEScan-non-LTR: computational identification and classification of autonomous non-LTR retrotransposons in eukaryotic genomes. Nucleic acids research. 2009;37 21:e143-e.
30. Crescente JM, Zavallo D, Helguera M and Vanzetti LS. MITE Tracker: an accurate approach to identify miniature inverted-repeat transposable elements in large genomes. BMC bioinformatics. 2018;19 1:348.
31. Xiong W, He L, Lai J, Dooner HK and Du C. HelitronScanner uncovers a large overlooked cache of Helitron transposons in many plant genomes. Proceedings of the National Academy of Sciences. 2014;111 28:10263-8.
32. Wicker T, Sabot F, Hua-Van A, Bennetzen JL, Capy P, Chalhoub B, et al. A unified classification system for eukaryotic transposable elements. Nat Rev Genet. 2007;8 12:973-82.
33. Jurka J, Kapitonov VV, Pavlicek A, Klonowski P, Kohany O and Walichiewicz J. Repbase Update, a database of eukaryotic repetitive elements. Cytogenetic and genome research. 2005;110 1-4:462-7.
34. RepeatMasker version 4.0.7. http://www.repeatmasker.org/. Accessed March 15 2020.
35. Luo M-C, Gu YQ, Puiu D, Wang H, Twardziok SO, Deal KR, et al. Genome sequence of the progenitor of the wheat D genome Aegilops tauschii. Nature. 2017;551 7681:498-502.
36. Andrews S: FastQC a quality control tool for high throughput sequence data. https://www bioinformatics babraham ac uk/projects/fastqc/. Accessed June 19 2020.
37. Bolger AM, Lohse M and Usadel B. Trimmomatic: a flexible trimmer for Illumina sequence data. Bioinformatics (Oxford, England). 2014;30 15:2114-20.
38. Kim D, Langmead B and Salzberg SL. HISAT: a fast spliced aligner with low memory requirements. Nature methods. 2015;12 4:357-60.

39. Pertea M, Pertea GM, Antonescu CM, Chang T-C, Mendell JT and Salzberg SL. StringTie enables improved reconstruction of a transcriptome from RNA-seq reads. Nature biotechnology. 2015;33 3:290-5.
40. Haas BJ, Papanicolaou A, Yassour M, Grabherr M, Blood PD, Bowden J, et al. De novo transcript sequence reconstruction from RNA-seq using the Trinity platform for reference generation and analysis. Nature protocols. 2013;8 8:1494-512.
41. Campbell MS, Law M, Holt C, Stein JC, Moghe GD, Hufnagel DE, et al. MAKER-P: a tool kit for the rapid creation, management, and quality control of plant genome annotations. Plant physiology. 2014;164 2:513-24.
42. Stanke M, Steinkamp R, Waack S and Morgenstern B. AUGUSTUS: a web server for gene finding in eukaryotes. Nucleic acids research. 2004;32 suppl_2:W309-W12.
43. Korf I. Gene finding in novel genomes. BMC bioinformatics. 2004;5 1:59.
44. Borodovsky M, Mills R, Besemer J and Lomsadze A. Prokaryotic gene prediction using GeneMark and GeneMark. hmm. Current protocols in bioinformatics. 2003;1 1:4.5. 1-4.5. 16.
45. Simao Neto F, Waterhouse R, Ioannidis P, Kriventseva E and Zdobnov E. BUSCO: assessing genome assembly and annotation completeness with single-copy orthologs. Bioinformatics (Oxford, England). 2015;31 19:3210-2.
46. Van Bel M, Diels T, Vancaester E, Kreft L, Botzki A, Van de Peer Y, et al. PLAZA 4.0: an integrative resource for functional, evolutionary and comparative plant genomics. Nucleic acids research. 2018;46 D1:D1190-D6.
47. Campbell MS, Holt C, Moore B and Yandell M. Genome annotation and curation using MAKER and MAKER‐P. Current protocols in bioinformatics. 2014;48 1:4.11. 1-4.. 39.
48. Altschul SF, Madden TL, Schäffer AA, Zhang J, Zhang Z, Miller W, et al. Gapped BLAST and PSI-BLAST: a new generation of protein database search programs. Nucleic acids research. 1997;25 17:3389-402.
49. Jones P, Binns D, Chang H-Y, Fraser M, Li W, McAnulla C, et al. InterProScan 5: genome-scale protein function classification. Bioinformatics (Oxford, England). 2014;30 9:1236-40.
50. Waterhouse RM, Seppey M, Simão FA, Manni M, Ioannidis P, Klioutchnikov G, et al. BUSCO applications from quality assessments to gene prediction and phylogenomics. Molecular biology and evolution. 2018;35 3:543-8.
51. Nishimura O, Hara Y and Kuraku S. gVolante for standardizing completeness assessment of genome and transcriptome assemblies. Bioinformatics (Oxford, England). 2017;33 22:3635-7.
52. Nawrocki EP and Eddy SR. Infernal 1.1: 100-fold faster RNA homology searches. Bioinformatics (Oxford, England). 2013;29 22:2933-5.
53. Kalvari I, Argasinska J, Quinones-Olvera N, Nawrocki EP, Rivas E, Eddy SR, et al. Rfam 13.0: shifting to a genome-centric resource for non-coding RNA families. Nucleic acids research. 2018;46 D1:D335-D42.
54. Kalvari I, Nawrocki EP, Argasinska J, Quinones‐Olvera N, Finn RD, Bateman A, et al. Non‐coding RNA analysis using the Rfam database. Current protocols in bioinformatics. 2018;62 1:e51.
55. Dongen S. *Graph clustering by flow simulation*. University of Utrecht, Amsterdam, Netherlands, 2000.

56. Katoh K and Standley DM. MAFFT multiple sequence alignment software version 7: improvements in performance and usability. Molecular biology and evolution. 2013;30 4:772-80.
57. Yang Z. PAML 4: phylogenetic analysis by maximum likelihood. Molecular biology and evolution. 2007;24 8:1586-91.
58. Price MN, Dehal PS and Arkin AP. FastTree 2–approximately maximum-likelihood trees for large alignments. PloS one. 2010;5 3:e9490.
59. Proost S, Fostier J, De Witte D, Dhoedt B, Demeester P, Van de Peer Y, et al. i-ADHoRe 3.0—fast and sensitive detection of genomic homology in extremely large data sets. Nucleic acids research. 2012;40 2:e11-e.
60. Emms DM and Kelly S. OrthoFinder: phylogenetic orthology inference for comparative genomics. Genome biology. 2019;20 1:1-14.
61. Whelan S, Irisarri I and Burki F. PREQUAL: detecting non-homologous characters in sets of unaligned homologous sequences. Bioinformatics (Oxford, England). 2018;34 22:3929-30.
62. Ronquist F, Teslenko M, Van Der Mark P, Ayres DL, Darling A, Höhna S, et al. MrBayes 3.2: efficient Bayesian phylogenetic inference and model choice across a large model space. Systematic biology. 2012;61 3:539-42.
63. Rannala B and Yang Z. Inferring speciation times under an episodic molecular clock. Systematic biology. 2007;56 3:453-66.
64. Iles WJ, Smith SY, Gandolfo MA and Graham SW. Monocot fossils suitable for molecular dating analyses. Bot J Linn Soc. 2015;178 3:346-74.
65. Kumar S, Stecher G, Suleski M and Hedges SB. TimeTree: a resource for timelines, timetrees, and divergence times. Molecular biology and evolution. 2017;34 7:1812-9.
66. Bennetzen JL and Park M. Distinguishing friends, foes, and freeloaders in giant genomes. Curr Opin Genet Dev. 2018;49:49-55.
67. Schnable PS, Ware D, Fulton RS, Stein JC, Wei F, Pasternak S, et al. The B73 maize genome: complexity, diversity, and dynamics. science. 2009;326 5956:1112-5.
68. Paterson AH, Bowers JE, Bruggmann R, Dubchak I, Grimwood J, Gundlach H, et al. The Sorghum bicolor genome and the diversification of grasses. Nature. 2009;457 7229:551-6.
69. Bennetzen JL and Wang H. The contributions of transposable elements to the structure, function, and evolution of plant genomes. Annual review of plant biology. 2014;65:505-30.
70. Devos KM, Brown JK and Bennetzen JL. Genome size reduction through illegitimate recombination counteracts genome expansion in Arabidopsis. Genome research. 2002;12 7:1075-9.
71. Ma J, Devos KM and Bennetzen JL. Analyses of LTR-retrotransposon structures reveal recent and rapid genomic DNA loss in rice. Genome research. 2004;14 5:860-9.
72. Jiao Y, Li J, Tang H and Paterson AH. Integrated syntenic and phylogenomic analyses reveal an ancient genome duplication in monocots. The Plant cell. 2014;26 7:2792-802.
73. Abdul SD and Jideani AIO. Fonio (Digitaria spp.) Breeding. Springer International Publishing; 2019. p. 47-81.
74. Soltis D, Soltis P and Rieseberg LH. Molecular data and the dynamic nature of polyploidy. Critical reviews in plant sciences. 1993;12 3:243-73.

75. Sybenga J. Allopolyploidization of autopolyploids I. Possibilities and limitations. Euphytica. 1969;18 3:355-71.
76. Bird KA, VanBuren R, Puzey JR and Edger PP. The causes and consequences of subgenome dominance in hybrids and recent polyploids. New Phytologist. 2018;220 1:87-93.
77. Lin Z, Li X, Shannon LM, Yeh C-T, Wang ML, Bai G, et al. Parallel domestication of the Shattering1 genes in cereals. Nature genetics. 2012;44 6:720-4.
78. Geneious Prime. https://www.geneious.com. 2020.
79. Multani DS, Briggs SP, Chamberlin MA, Blakeslee JJ, Murphy AS and Johal GS. Loss of an MDR transporter in compact stalks of maize br2 and sorghum dw3 mutants. Science. 2003;302 5642:81-4.
80. Parvathaneni RK, Jakkula V, Padi FK, Faure S, Nagarajappa N, Pontaroli AC, et al. Fine-mapping and identification of a candidate gene underlying the d2 dwarfing phenotype in pearl millet, Cenchrus americanus (L.) Morrone. G3: Genes, Genomes, Genetics. 2013;3 3:563-72.
81. Simmonds J, Scott P, Brinton J, Mestre TC, Bush M, Del Blanco A, et al. A splice acceptor site mutation in TaGW2-A1 increases thousand grain weight in tetraploid and hexaploid wheat through wider and longer grains. Theoretical and Applied Genetics. 2016;129 6:1099-112.
82. Song X-J, Huang W, Shi M, Zhu M-Z and Lin H-X. A QTL for rice grain width and weight encodes a previously unknown RING-type E3 ubiquitin ligase. Nature genetics. 2007;39 5:623-30.
83. Wu TD, Reeder J, Lawrence M, Becker G and Brauer MJ. GMAP and GSNAP for genomic sequence alignment: enhancements to speed, accuracy, and functionality. Statistical genomics. Springer; 2016. p. 283-334.
84. Li H. A statistical framework for SNP calling, mutation discovery, association mapping and population genetical parameter estimation from sequencing data. Bioinformatics (Oxford, England). 2011;27 21:2987-93.
85. Bradbury PJ, Zhang Z, Kroon DE, Casstevens TM, Ramdoss Y and Buckler ES. TASSEL: software for association mapping of complex traits in diverse samples. Bioinformatics (Oxford, England). 2007;23 19:2633-5.
86. Kahle D and Wickham H. ggmap: Spatial Visualization with ggplot2. The R journal. 2013;5 1:144-61.
87. Raj A, Stephens M and Pritchard JK. fastSTRUCTURE: variational inference of population structure in large SNP data sets. Genetics. 2014;197 2:573-89.
88. Zhu Q, Smith SM, Ayele M, Yang L, Jogi A, Chaluvadi SR, et al. High-throughput discovery of mutations in tef semi-dwarfing genes by next-generation sequencing analysis. Genetics. 2012;192 3:819-29.
89. Ntui VO, Azadi P, Supaporn H and Mii M. Plant regeneration from stem segment-derived friable callus of “Fonio”(Digitaria exilis (L.) Stapf.). Scientia horticulturae. 2010;125 3:494-9.
90. Ji X, Yang B and Wang D. Achieving Plant Genome Editing While Bypassing Tissue Culture. Trends Plant Sci. 2020.
91. Hu N, Xian Z, Li N, Liu Y, Huang W, Yan F, et al. Rapid and user-friendly open-source CRISPR/Cas9 system for single-or multi-site editing of tomato genome. Horticulture research. 2019;6 1:1-14.

92. Sterck L, Billiau K, Abeel T, Rouze P and Van de Peer Y. ORCAE: online resource for community annotation of eukaryotes. Nature Methods. 2012;9 11:1041-.
93. Yssel AE, Kao S-M, Van de Peer Y and Sterck L. ORCAE-AOCC: A Centralized Portal for the Annotation of African Orphan Crop Genomes. Genes. 2019;10 12:950.
94. Yves Van De Peer: Orcae: Online Resource for Community Annotation of Eukarytotes. https://bioinformatics.psb.ugent.be/orcae/aocc/overview/Digex) (2020). Accessed June 21 2020.
95. Wallace J: Fonio diveristy 2020. https://github.com/wallacelab/paper-fonio-diversity-2020 (2020). Accessed June 21 2020.
96. Bennetzen JL and Chen S; Ma X WX, Yssel AEJ, Chaluvadi SR, Johnson MS, Gangashetty P, Hamidou F, Sanogo MD, Zwaenepoel A, Wallace J, Van De Peer Y, Van Deynze A Supporting data for "Genome sequence and genetic diversity analysis of an under-domesticated orphan crop, white fonio (*Digitaria exilis*)" GigaScience Database, 2021. http://doi.org/10.5524/100857.

Figure 1

Click here to access/download;Figure;Figure 1.pdf

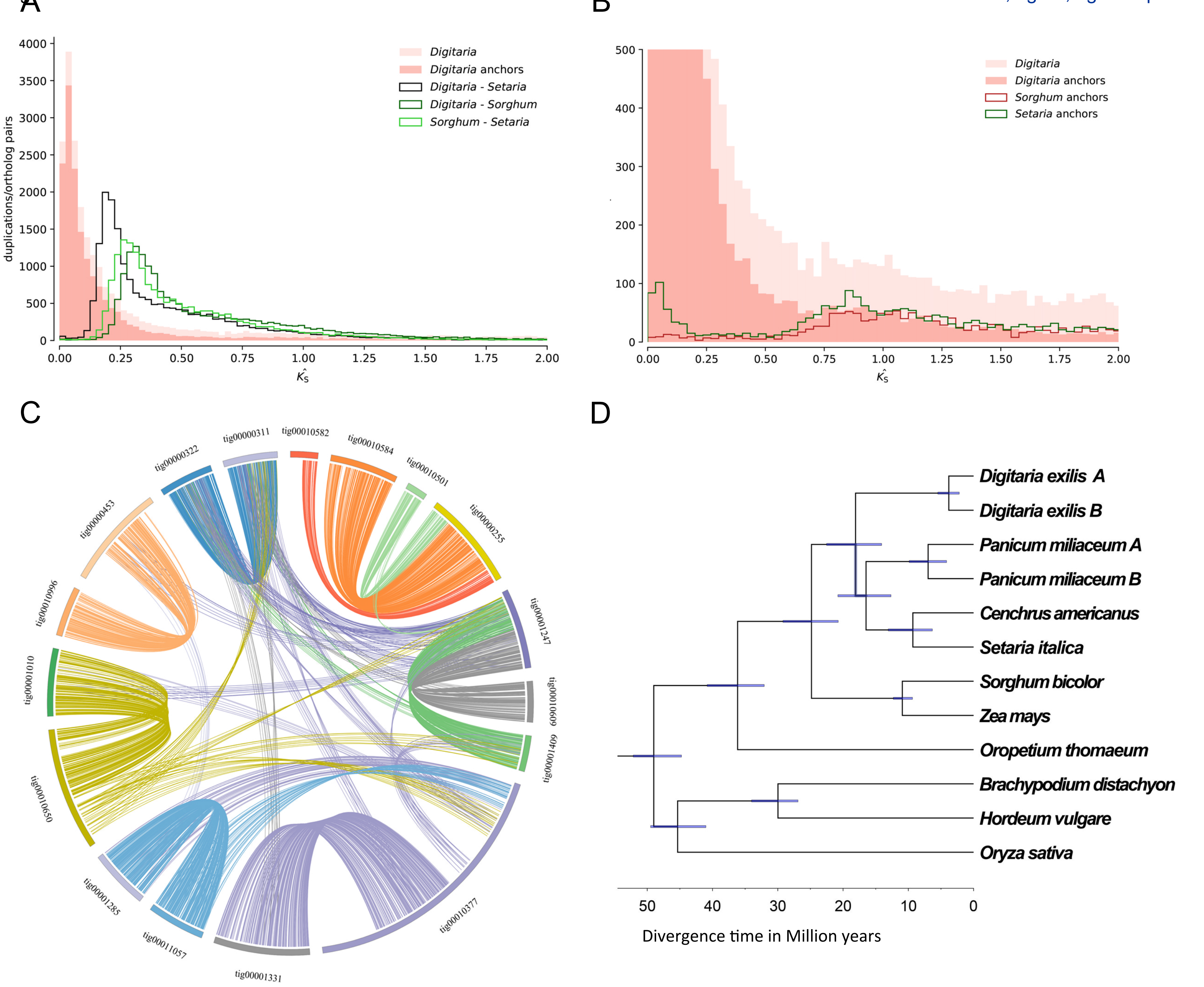

Figure 2 Click here to access/download;Figure;Figure 2.docx

Figure 2

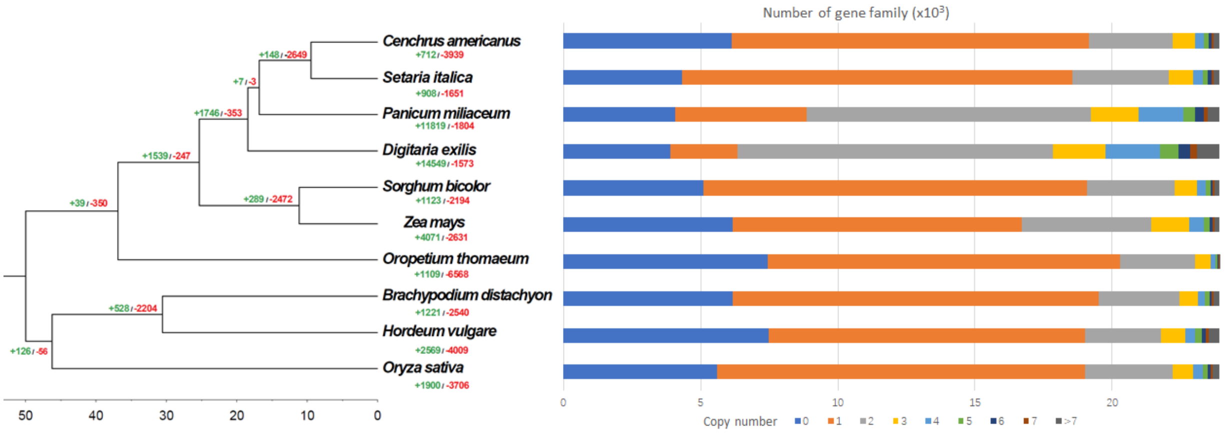

*green numbers represent genes with greater than 2 copies and red numbers gens with less than 2 copies

Supplementary Methods

Click here to access/download
Supplementary Material
Supplementary Methods 122020.docx

Click here to access/download
Supplementary Material
Suppl. Tables and Figures 01192021.docx

Click here to access/download
Supplementary Material
Fonio Suppl Table S7and S8.xlsx

Response to Review Click here to access/download;Personal Cover;Fonio Response to review-12112020.docx

Response to review GIGA-D-20-00197

Dear Editor,

We wish to thank the reviewers for their many thoughtful comments and suggestions. We address all of the issues raised in our replies below, which are in blue typeface. We believe that the additional analyses and revisions that we have made, associated with the reviewers' comments, have significantly improved the manuscript. Please find tracked changes version and clean version.

Sincerely,
Jeff Bennetzen for all authors

Comments and responses:

Reviewer 1

1. The original manuscript seemed to a well oral draft for speaker, especially in the sections of background, plant material and conclusions. Thanks you
2. In the section of Plant material and nucleic preparation, please provide the original source of fonio seeds and its latitude-longitude The contents and their proportions of standard potting soil should be indicated. Thank you for the suggestion. We have added this information to Plant materials.
3. In the logically, estimation of genome and heterozygosity with illumina reads were before assembly of PacBio reads and polished with illumine reads. Please consider, this is not must revised if it were not necessary. Thank you for the suggestion. Using raw unassembled reads and kmer analysis is one measure of heterozygosity in polyploid species as referenced by Ranallo-Benavidez, T.R., K.S. Jaron, and M.C. Schatz. 2020. GenomeScope 2.0 and Smudgeplot for reference-free profiling of polyploid genomes. Nature Communications 11:1432, https://doi.org/10.1038/s41467-020-14998-3. Genoscope is a tool specifically designed to estimate heterozygosity in polyploids. We have added this reference.

4. Repeat annotation and TE properties are continuous. In the section of TE properties, average size of Gypsy is the double of Copia, why? Although, we do not have a conclusive answer and, we noted this as well. we discuss this on lines 311-318 in text.
5. For more intuitively, please add the most recent common ancestor and predicted divergence time or confidence interval beside the node in Fig1D. The analysis presented in Figure 1D uses the most closely related species to fonio that have genome sequences, and all are millets. Namely *P. millaceum*, *S. italica* and *C. americanus*. The diploid ancestors of fonio is not clear (Abdul et al 2019) and highly likely to be extinct considering the estimated time of polyploidization 3.1MYA. We added text on these points to lines 339-341.
6. I noticed that KAUST has been upload their genome of D.exilis on NCBI with the BioProject number: PRJEB36539. The quality of assembly sequence in this manuscript is much higher than theirs. Comparing or mentioning their assembly will look fair and highlight the higher quality assembly of the genome you present here. Thank you for the suggestion. This paper has been published since our submission. We added comparison tables (Suppl 1) and Suppl Fig 1 as well as text on page 5. We show that our genome is in significantly better shape, for instance with an L50 of 8 vs 2624, with more of the genome assembled and better BUSCO score and complete

genes annotated, as mentioned on pages 5 and 8. We further compare a typical scaffold in our assembly and show the fragmentation in the Abrouk assembly for the same genomic areas. Some aspects of scaffolding are better in the Abrouk et al genome due to the use of scaffolding technology, Hi-C.

7. The candidate domestication genes were well aligned and discussed. But in the abstract, resilience in hot, dry and low fertility environments of *D.exilis* were highlighted. Have you found special gene families related with these physiological features in your analysis? Excellent resources of resistance gene or TFs are also important for genetic improvement of other cereal crops. Expansion and contraction of gene family might provide some preliminary clues. We added a section on gene family expansion and contraction, including GO enrichments noting emphasis on recognition motifs. See page 13. We do not expand our Discussion of the many hundreds of possible resilience genes *per se*, because we did not perform any experiments related to this subject.

8. RRID numbers were not contained in this original manuscript. We have not created any new softwares or software packages for analyses of these data. All software packages used were “off the shelf” and are described in the MS and supplemental methods. For this reason no new RRID numbers have been generated.

Reviewer 2

Specific comments:

Line 44: Precise in the abstract what genes and traits could be used for fonio Improvement. We have now added text about the genes discussed. Thank you for this suggestion.

Line 97: Revise the Plant material and nucleic acid preparation about Genetic Diversity for Nagoya protocol). Summarize the protocol focusing on fonio. The authors should be more specific See addition in Plant materials and to Genetic diversity lines 450-452.

Line 99: A plant of fonio would be valuable to put in as it is an orphan crop. Not a common plant. The authors can add an additional figure with scale for plant, seeds. Although the authors agree and point out that it is an orphan crop, there are many pictures online of fonio, its seed, etc. For example, see https://en.wikipedia.org/wiki/Fonio. We respectfully did not add this to the revised MS.

Line 104: Describe in detail the method used for DNA isolation rather than giving the reference. This is important for quality control and orphan crops. A brief description of extraction was added, see Lines 105-116

Line 113: Give references for SMRT Link and Canu (v1.8). We added references to these software packages. Thanks for pointing out this omission.

Line 115: Pilon (v 1.23) : remove space Done.

Line 118: The longest contig is 10.17 Mb and the shortest contig is 1013 bp: Give the average contig. The mean value has been added to this line, now line 133.

Line 131-133: What is your hypothesis about the reduction of the genome size of 200 Mb? That is a large portion of the genome not captured. Provide explanations See lines 160-162.

Line 209: Summarize the protocol here or add the parameters you used. Respectfully, as we used the protocol exactly as defined in Kalvari et al., we defer to the reference.

Line 209: This sentence does not make sense for non-coding RNAs, it will be better if the authors add the percentage of non-coding RNAs types within the 4741 RNAs. Also,

add citations for the other plant genomes. We have added the percentage of RNAs in text (Line 246) and also to Supplement Table 3.
Line 224: Repetition Plaza Also, this sentence does not make sense. We have revised for clarity, so now see line 261.
Line 240: There is an extra parenthesis Corrected.
Line 299: The hypothesis for major rearrangements on the genome is not well supported. Please provide more pieces of evidence. We have added a whole section on gene expansion and contraction to further discuss this point, including single copy genes rather duplicated genes expected in a tetraploid. Note, although we do not have a chromosome level assembly (see Reviewer 1, question 6) we show very high contiguity of the genome to accurately assess the rearrangements and synteny Suppl Fig S1, and Suppl Fig S4.
Line 323: Shattering genes are essential in fonio since the farmers harvest before the maturity to limit yield loss. The figures shouldn't appear in the supplemental information to highlight its importance. Although we agree, this is one of several important examples. The MS already has many figures and tables highlighting broad analyses. For this reason, we chose to keep the figure at a supplemental level.
Line 369: For crops like fonio, the threshold for heterozygosity that the author used is very conservative. Have the authors tried <10% and 15%? We performed the analysis at several heterozygosity thresholds, and this value was the one that provided the most resolution of the fonio populations. At this high level of heterozygosity, it is certain that some of the differentiation is actually between paralogs, but these differences are lineage-specific (hence the high resolution between accessions), and thus assist us in the process of accessing differences and commonalities in the fonio germplasm that was analyzed.
Line 370: The three clusters should be discussed in deep instead of just saying that they correlate with the geographical map. This section needs revisions on its own. This discussion has been expanded, on lines 448-450.
Lines 377-383: This paragraph does not make sense.
This paragraph describes the diversity analyses methods and was included in Supplemental methods under Genetic Diversity Analysis.
Line 390: The authors did not provide evidence for day length dependence in mutations. The sentence is only meant to reflect that daylength-dependence is an additional example of an important domestication gene. Genetic mapping in many other crops have shown that allelic variation (i.e., natural mutation) at these day-length dependence genes is involved in crop domestication, but we do not have any way of showing which specific mutations would be particularly appropriate goals for improvement at such loci in fonio. Such determination is for future studies.

Reviewer 3

1. Two sequencing platforms were used in the study; PacBio and Illumina. Even though the assembly almost covers the estimated genome size, it is presented as 3333 contigs. What is the difficulty faced by the authors to construct it into chromosome level? Small genome size as well as the availability of long reads could make it more feasible to construct the chromosomes. Also, only 88% of the RNAseq reads could be aligned to genome, which shows the incompleteness of the assembly. How do the authors justify this? Please see reviewer 1 Question 6.

Response to review GIGA-D-20-00197

2. Line no. 83
"is some transcript sequence data [13] at NCBI." We have updated this reference.
Line No. 164.
"Illumina RNA sequencing data (paired-end 100 bp) of Digitaria exilis [13] were downloaded". Here the reference cited is wrong. We corrected the reference to Sarah et al. 2017

3. 67855 protein coding gene were identified from the assembly. This is quite a large number compared to other related plants. However, it is expected due to the allotetraploid nature of the plant and 4.3% single nucleotide variation was observed in between the sub- genomes. All analyzed domestication genes are duplicated in fonio compared to other related plants. Apart from the expected doubling, is there an expansion in any particular gene family? Thank you for the suggestion. Indeed, there are many. We compared expansion and contraction (single copy) relative to *O. sativa* genes for the full range of GO annotations in fonio and several other species. We added a section, Figure 2 and Suppl Figures S5 and S6 to address this important issue. This discussion begins on page 13.

4. Is there any gene unique to either of the sub- genomes? Although this is certainly an interesting point, our genome assembly is not at the chromosome level, so subgenomes cannot be comprehensively compared. We do shed light on the topic by identifying single copy genes, as discussed in previous question. These results are in page 13, Figure S5 and Figure 2

5. Please provide legends for Fig. S1- A and B. We believe that the reviewer misread the legend and is referring to Fig S2 A and B (and C) which have legends. Fig s1 is a single figure. (Note these are figures now called Suppl Fig S2 and S3).
