# Supplementary material for: Genome sequence and genetic diversity analysis of an under-domesticated orphan crop, white fonio (Digitaria exilis)
Source: Gigascience. 2021 Mar 12;10(3):giab013. doi: 10.1093/gigascience/giab013 (PMC7953496; doi:10.1093/gigascience/giab013)
Supplement: giab013_Supplemental_Files [file giab013_supplemental_files.zip › Suppl. Tables and Figures 01192021.docx]

**Supplementary Tables and Figures**

**Suppl. Table S1** Comparison of genome assembly statistics of Fonio.

| Parameters | CM05836 [20] | Niatia Genome |
| --- | --- | --- |
| Platform | Illumina, 10X Genomics, Hi-C | Pacific Biosciences |
| Sequencing depth | 321x | 99x |
| Genome size, Mb | 716.42 | 760.66 |
| Contig number | 29,115 | 3,329 |
| Contig N50, kb | 78 | 1,734 |
| Contig L50 | 2,624 | 8 |
| Protein coding genes | 59,844 | 67,855 |
| Mean gene length, bp | 2,531 | 2,656 |
| Mean CDS length, bp | 1,055 | 1,224 |
| Mean exon per gene | 4.6 | 4.8 |
| Mean exon length, bp | 230 | 254 |
| Mean intron length, bp | 405 | 376 |

**Suppl. Table S2.** Statistics for the gene annotation

| Nr of protein coding genes | 67,855 |
| --- | --- |
| Mean gene length (bp) | 2668.8 |
| Mean CDS length (bp) | 1228.0 |
| Mean nr exons per gene | 4.8 |
| Mean exon length (bp) | 253.9 |
| Mean intron length | 550.6 |

**Suppl. Table S3.** Annotated non-coding RNA genes.

| **Target name** | **Count** | **Percentage** |
| --- | --- | --- |
| 5_8S_rRNA | 32 | 0.67 |
| 5S_rRNA | 580 | 12.23 |
| ACEA_U3 | 9 | 0.19 |
| C4 | 1 | 0.02 |
| ctRNA_pND324 | 1 | 0.02 |
| enod40 | 6 | 0.13 |
| Histone3 | 10 | 0.21 |
| Intron_gpI | 9 | 0.19 |
| Intron_gpII | 195 | 4.11 |
| IRE_II | 1 | 0.02 |
| IsrR | 11 | 0.23 |
| LSU's | 278 | 5.86 |
| mir's | 350 | 7.38 |
| RNase_MRP | 2 | 0.04 |
| sno RNAs | 1109 | 23.39 |
| Spliceosomal snRNAs | 375 | 7.91 |
| SRP's | 36 | 0.76 |
| SSU_rRNA | 249 | 5.25 |
| TPP | 2 | 0.04 |
| tRNA | 1478 | 31.17 |
| tRNA-Sec | 7 | 0.15 |
| **TOTAL** | **4741** |  |

**Suppl. Table S4.** Orthologs for Suppression of Shattering1 genes

| **Species** | **# of SSH1 orthologues** |
| --- | --- |
| *Digitaria exilis* | 4 |
| *Brachypodium distachyon* | 2 |
| *Cenchrus americanus* | 2 |
| *Hordeum vulgare* | 1 |
| *Oropetium thomaeum* | 2 |
| *Oryza sativa* | 2 |
| *Setaria italica* | 2 |
| *Sorghum bicolor* | 3 |
| *Zea mays* | 2 |

**Suppl. Table S5.** Orthologs of Dwarf Gene-3

| **Species** | **# of *dw3* orthologues** |
| --- | --- |
| *Digitaria exilis* | 2 |
| *Brachypodium distachyon* | 1 |
| *Cenchrus americanus* | 1 |
| *Hordeum vulgare* | 1 |
| *Oropetium thomaeum* | 1 |
| *Oryza sativa* | 1 |
| *Setaria italica* | 1 |
| *Sorghum bicolor* | 1 |
| *Zea mays* | 1 |

**Suppl. Table S6.** Orthologs of Grain Weight-2 genes

| **Species** | **# of GW2 orthologues** |
| --- | --- |
| *Digitaria exilis* | 2 |
| *Brachypodium distachyon* | 1 |
| *Cenchrus americanus* | 1 |
| *Hordeum vulgare* | 1 |
| *Oropetium thomaeum* | 1 |
| *Oryza sativa* | 1 |
| *Setaria italica* | 1 |
| *Sorghum bicolor* | 1 |
| *Zea mays* | 2 |

**Suppl. Table S7.** Passport data for accessions and samples used for diversity study (see Suppl. Tables Excel file)

**Suppl. Table S8.** SNP database used for diversity study (see Suppl. Tables Excel file)

**A.**


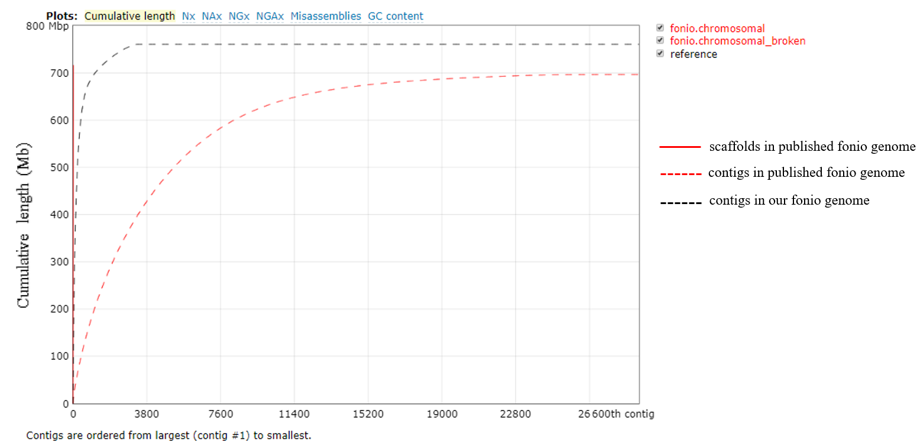

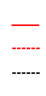


Scaffold CM05836

Contig CM05836

Contig Niatia

Contigs ordered from largest to smallest

Cumulative length (Mb)

**B.**


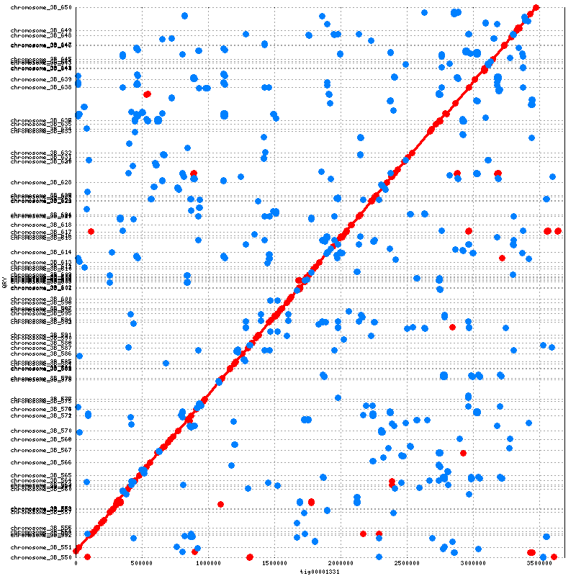

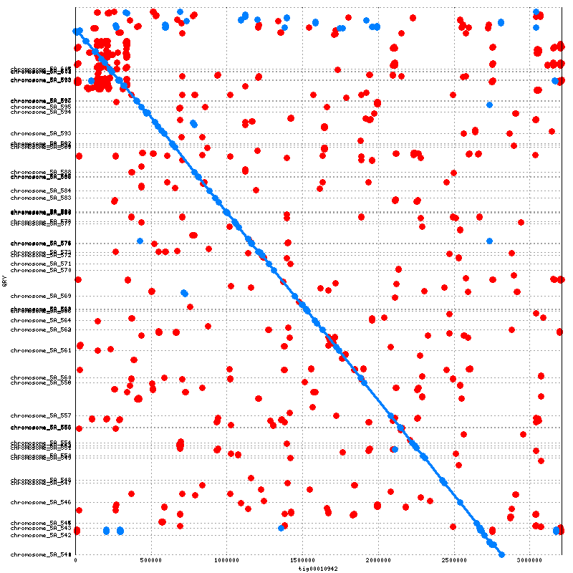


**Suppl Figure S1.**  **A**. comparison of the contiguity of the Niatia Genome and CM05836 [20] genome. **B**. comparison of Contig tig00001331 corresponding to 100 consecutive segments anchored on the same chromosome 3B and tig00010942 corresponding to 65 consecutive segments on the chromosome 5A on the Abrouk et al. [20] genome.


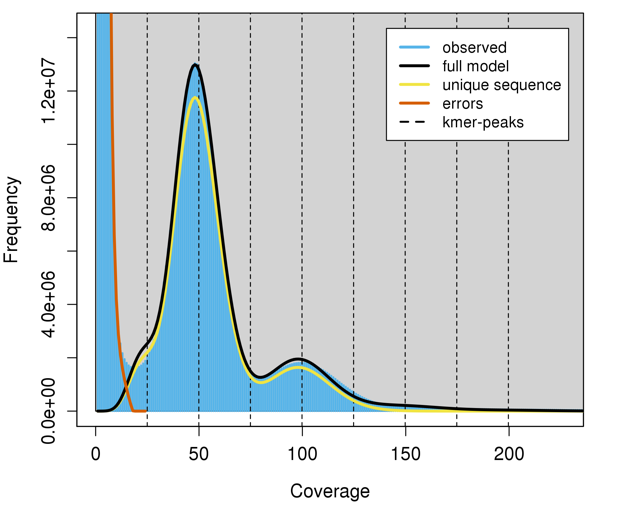


**Suppl. Figure S2.** The kmer distribution of raw Illumina reads at kmer value 33bp.

A


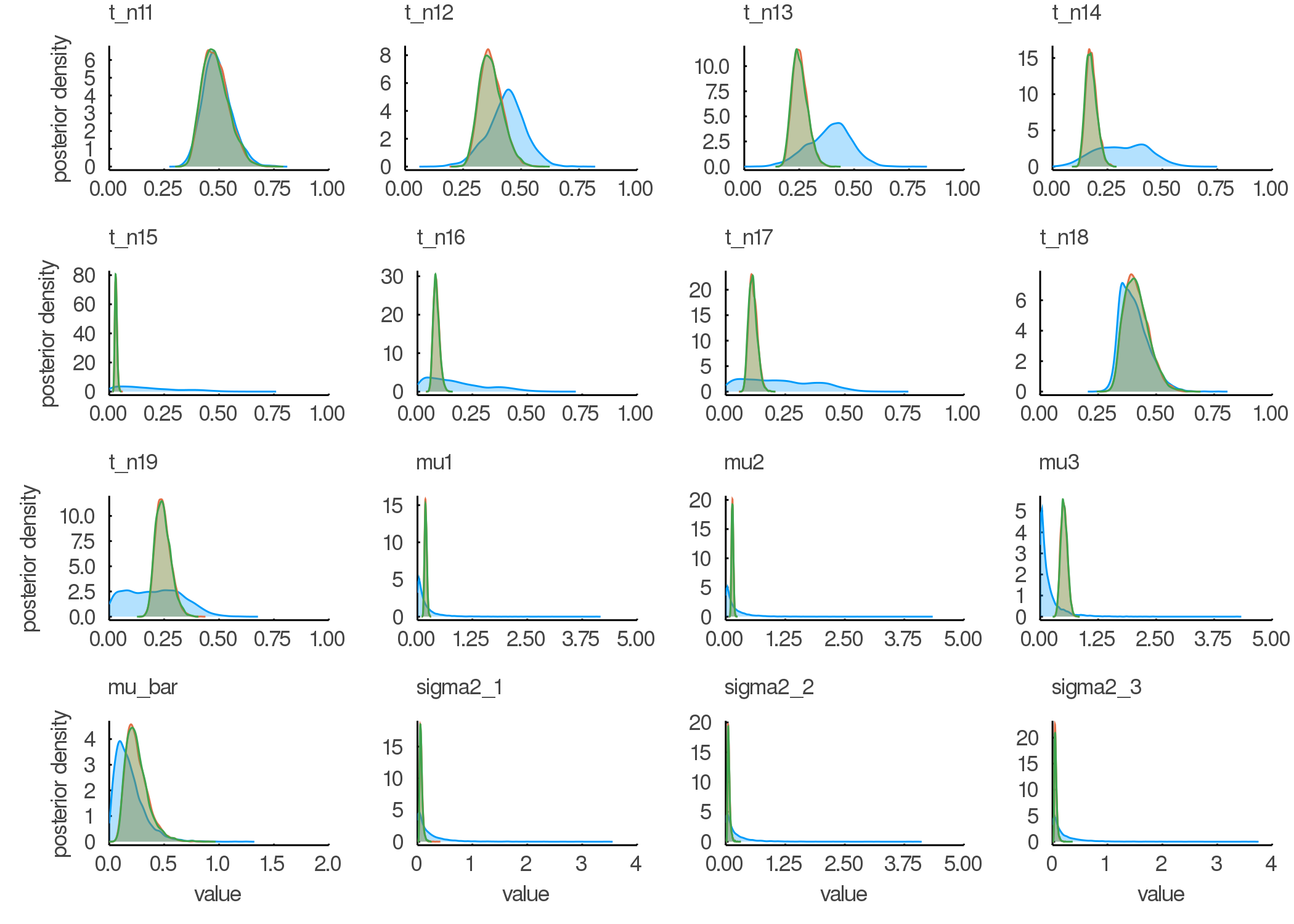


B


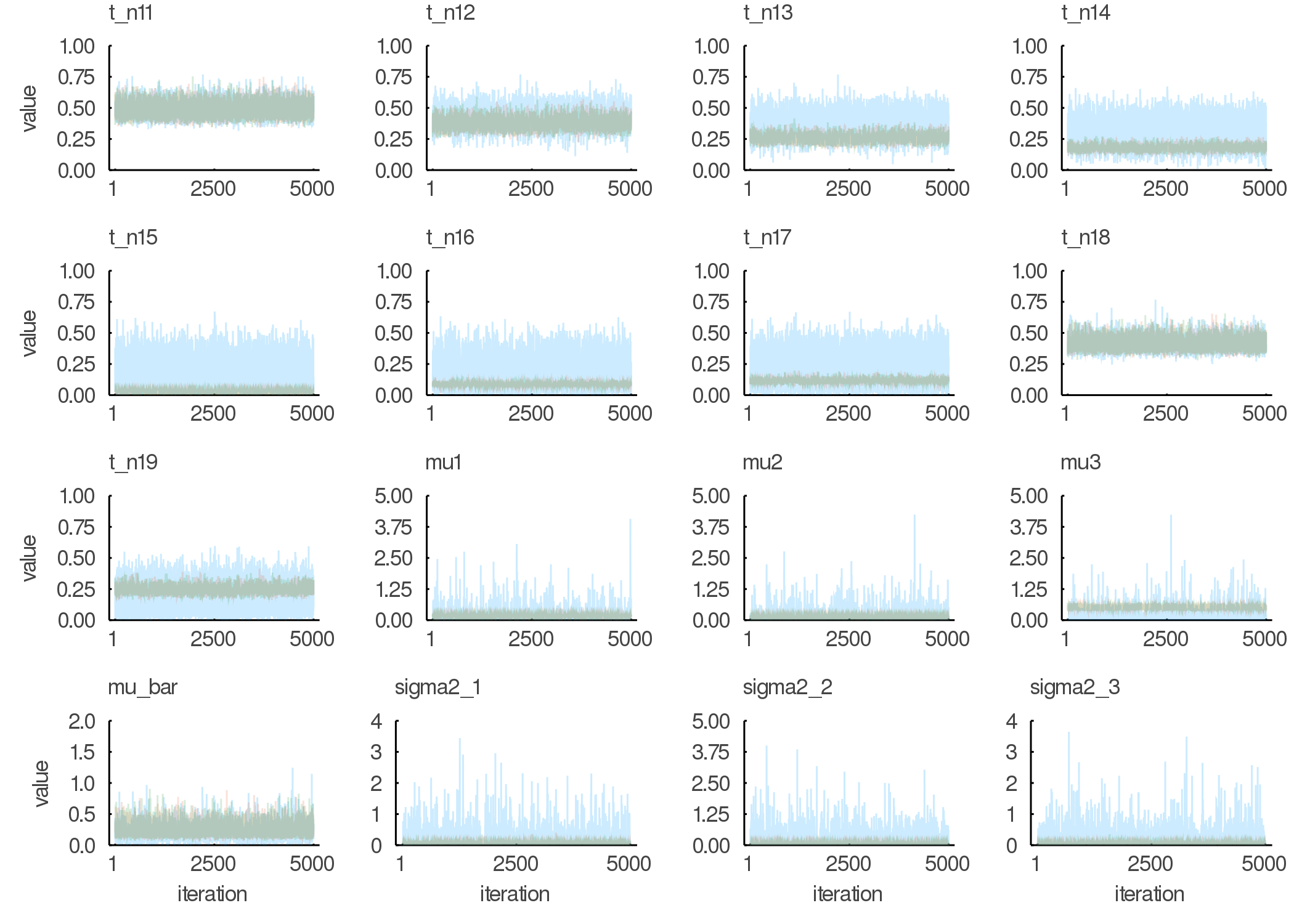


C


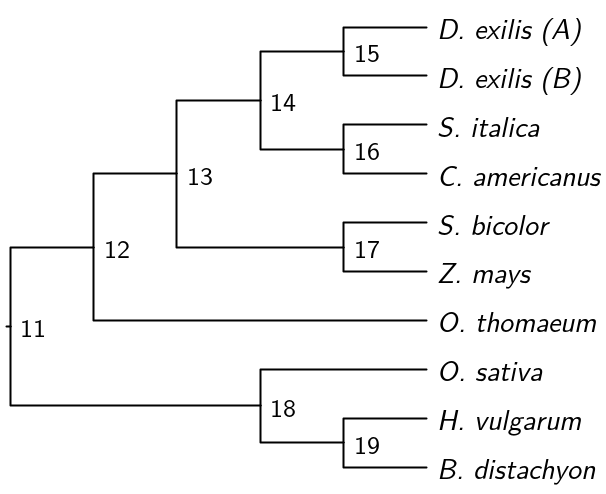


**Suppl. Figure S3.** **A** Marginal posterior distributions for two independent chains (green and orange) and induced marginal prior distributions (blue) for internal node ages (t_n11 to t_n19, see panel **C**), overall mean substitution rate (mu), mean substitution rate for different codon positions (mu1, mu2 and mu3) and variance parameter of the uncorrelated relaxed clock (sigma2_1, sigma2_2 and sigma2_3) for the three codon positions. **B** Trace plots for the MCMC chains associated with panel (**A**).


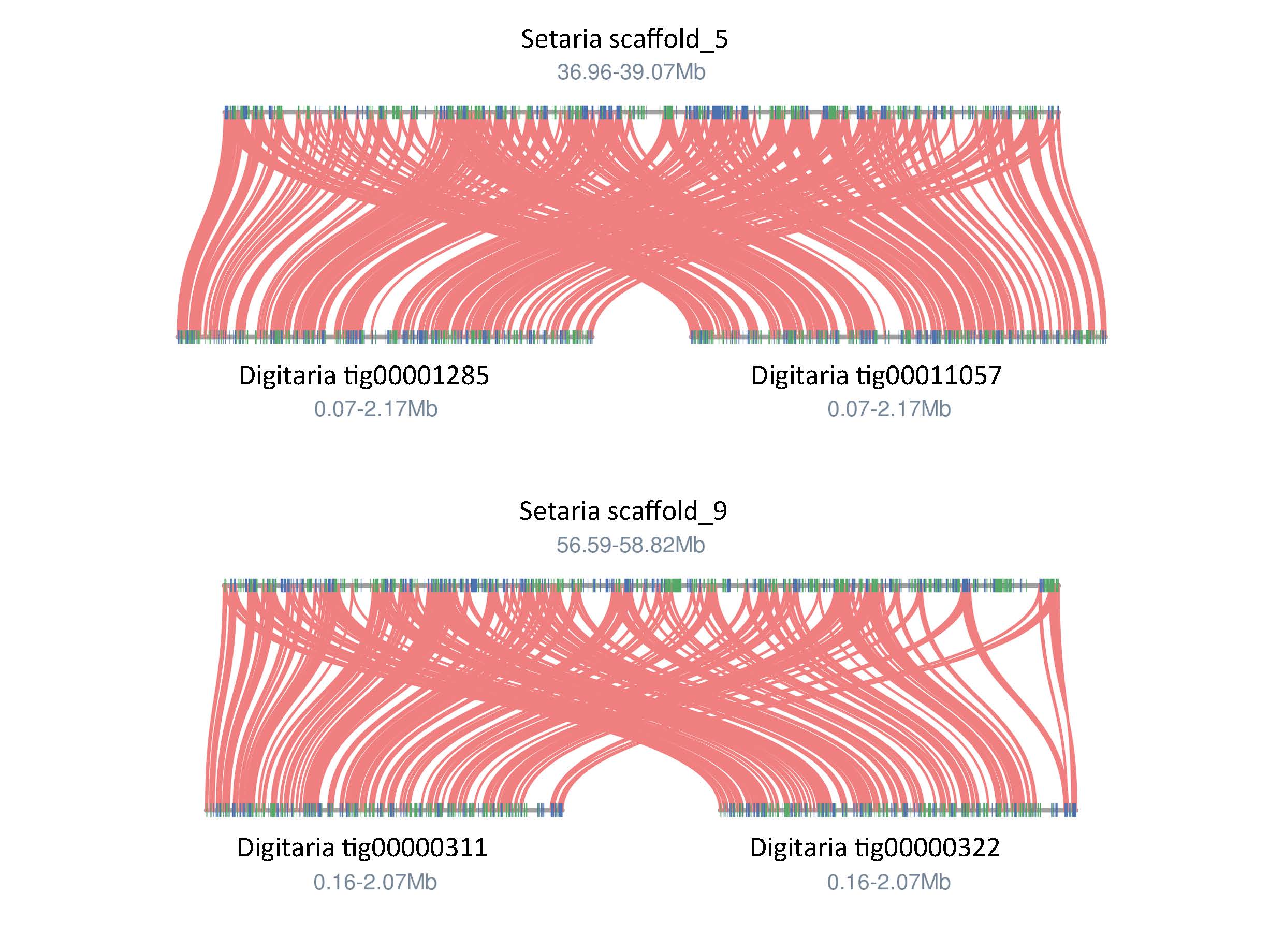


**Suppl Figure S4** There are 10075 families that have two-copy in fonio and one copy in *Setaria talica* and 90% of two-copy families are located in synteny blocks. The above four examples indicate the high degree of collinearity and synteny between *Setaria italica* and fonio.

**
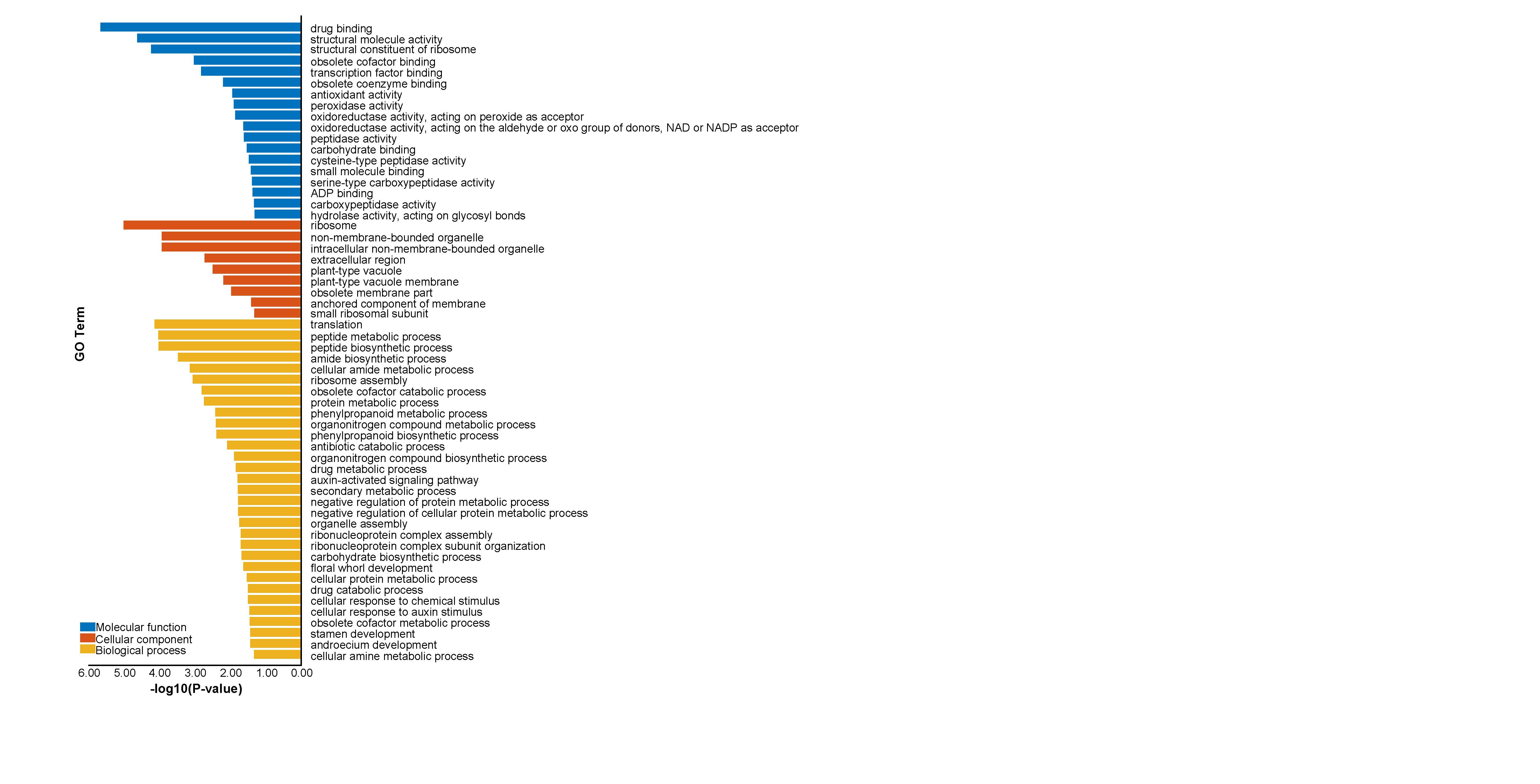
**

**Suppl Figure S5.** GO for of single copy, contracted genes in fonio.


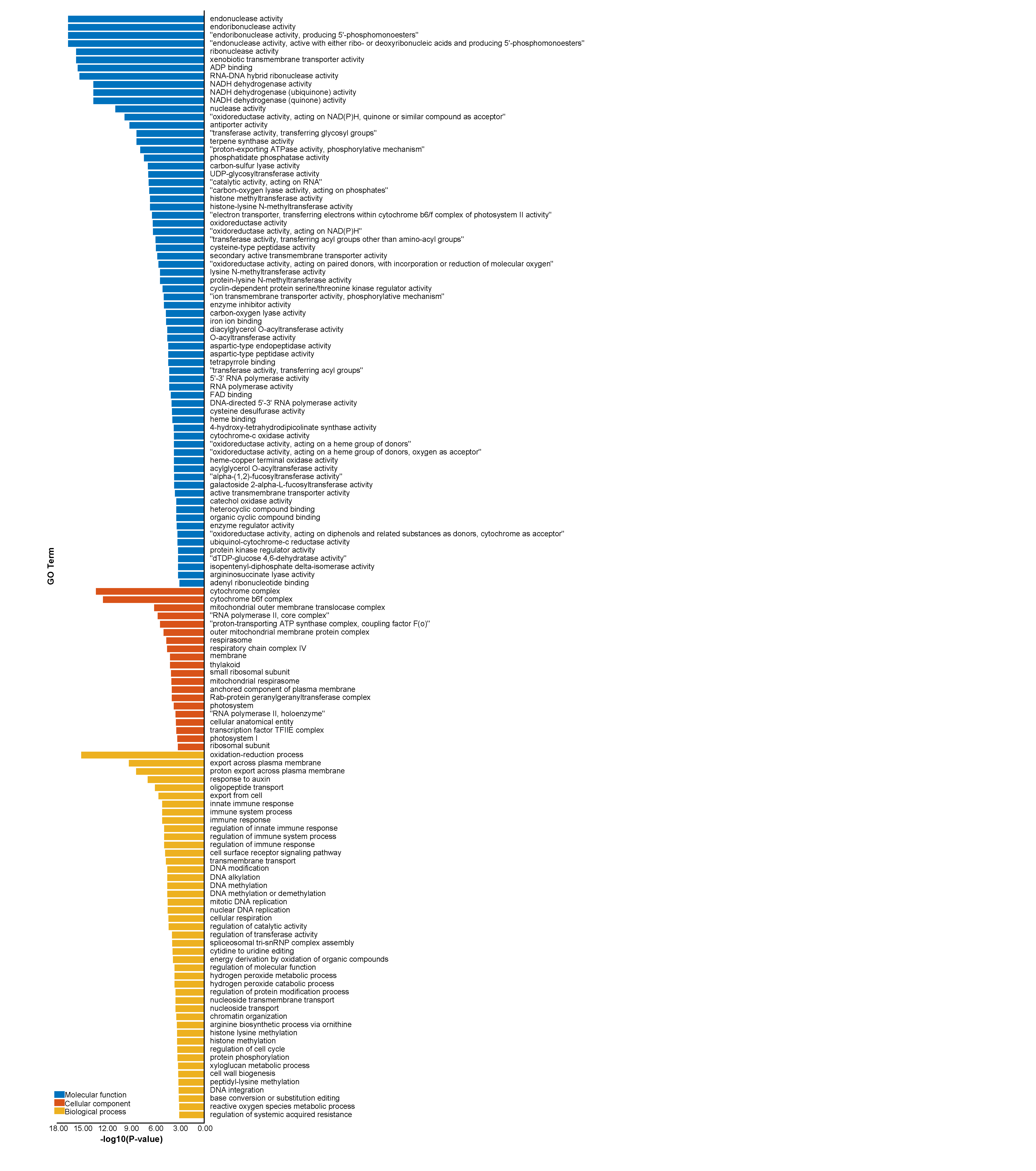


**Suppl Figure S6.** GO enrichment for expanded genes in *D. exilis* and relative to *O. sativa*


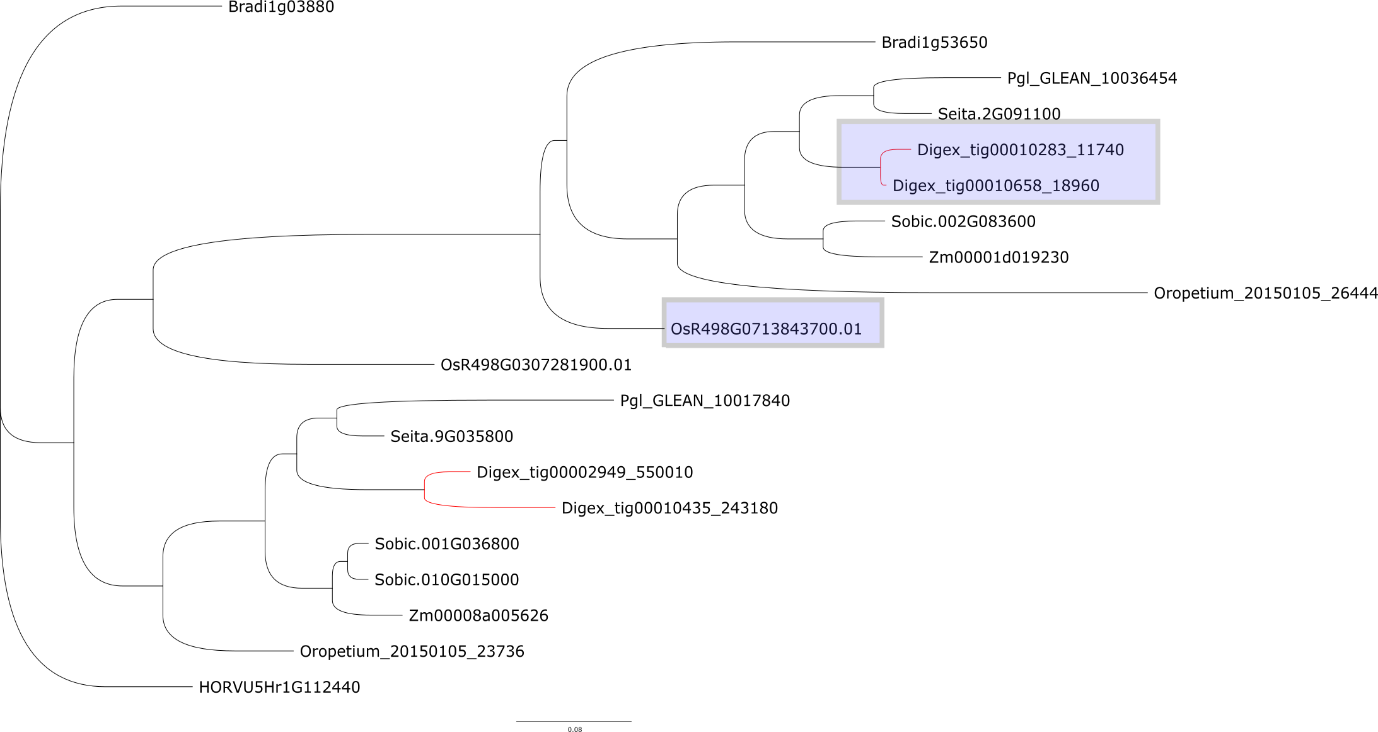


**Suppl. Figure S7.** Phylogenetic tree of the SSH-like genes from fonio and related species. The genes shaded in light blue are the family members most closely related to SSH-1 in O. sativa and D. exilis. Genes are named according to their PLAZA identifiers. Abbreviations for species names are as follow: Bradi (Brachypodium distachyon), pgl_GLEAN (Cenchrus amercianus), Digex (Digitaria exilis), Oropetium (Oropetium thomaeium), OsR (Oryza sativa), Seita (Setaria italica), Sobic (Sorghum bicolor), and Zm (Zea mays).


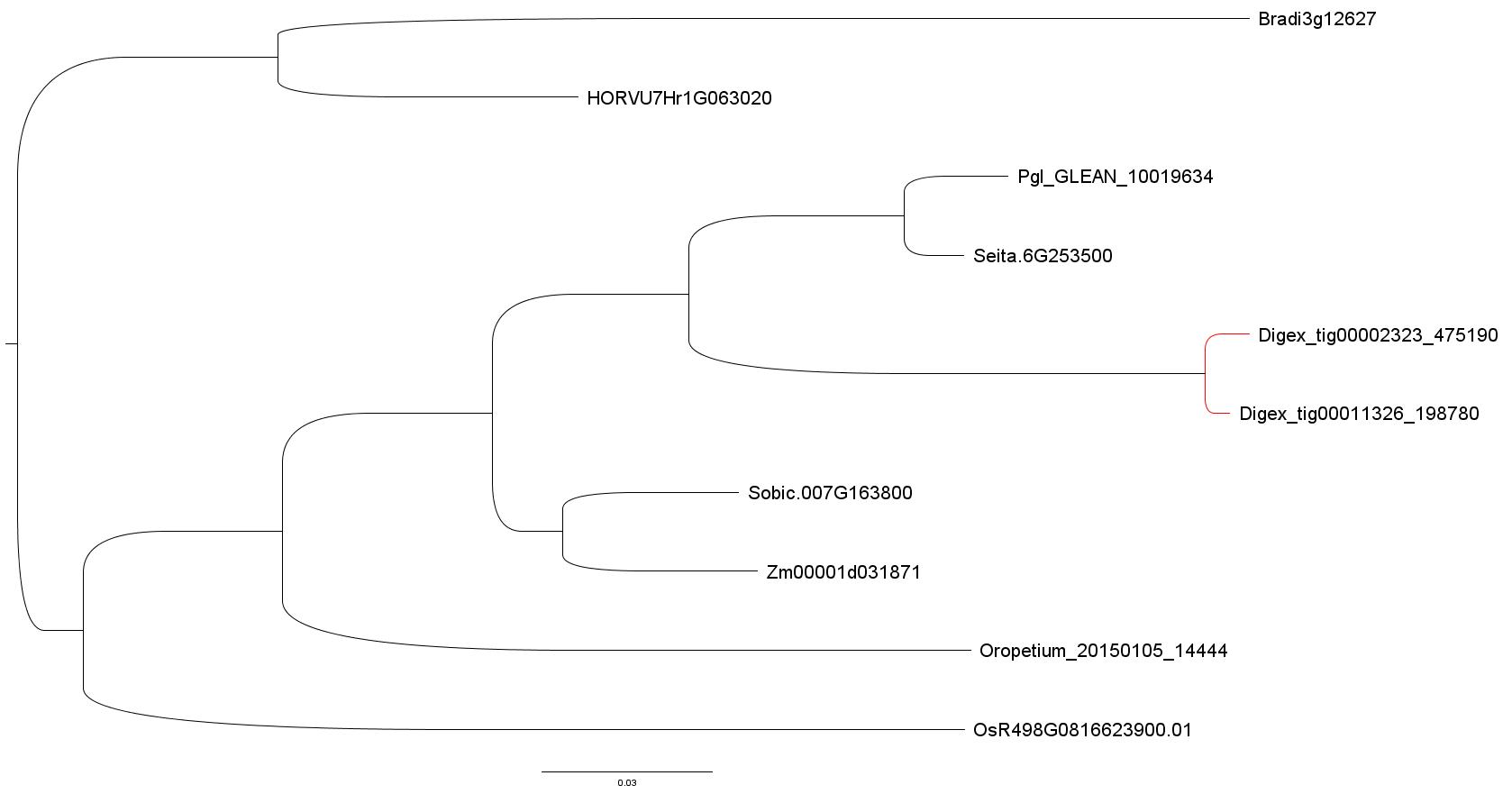


**Suppl. Figure S8.** Phylogenetic tree of the dw3 gene family of fonio and related species.


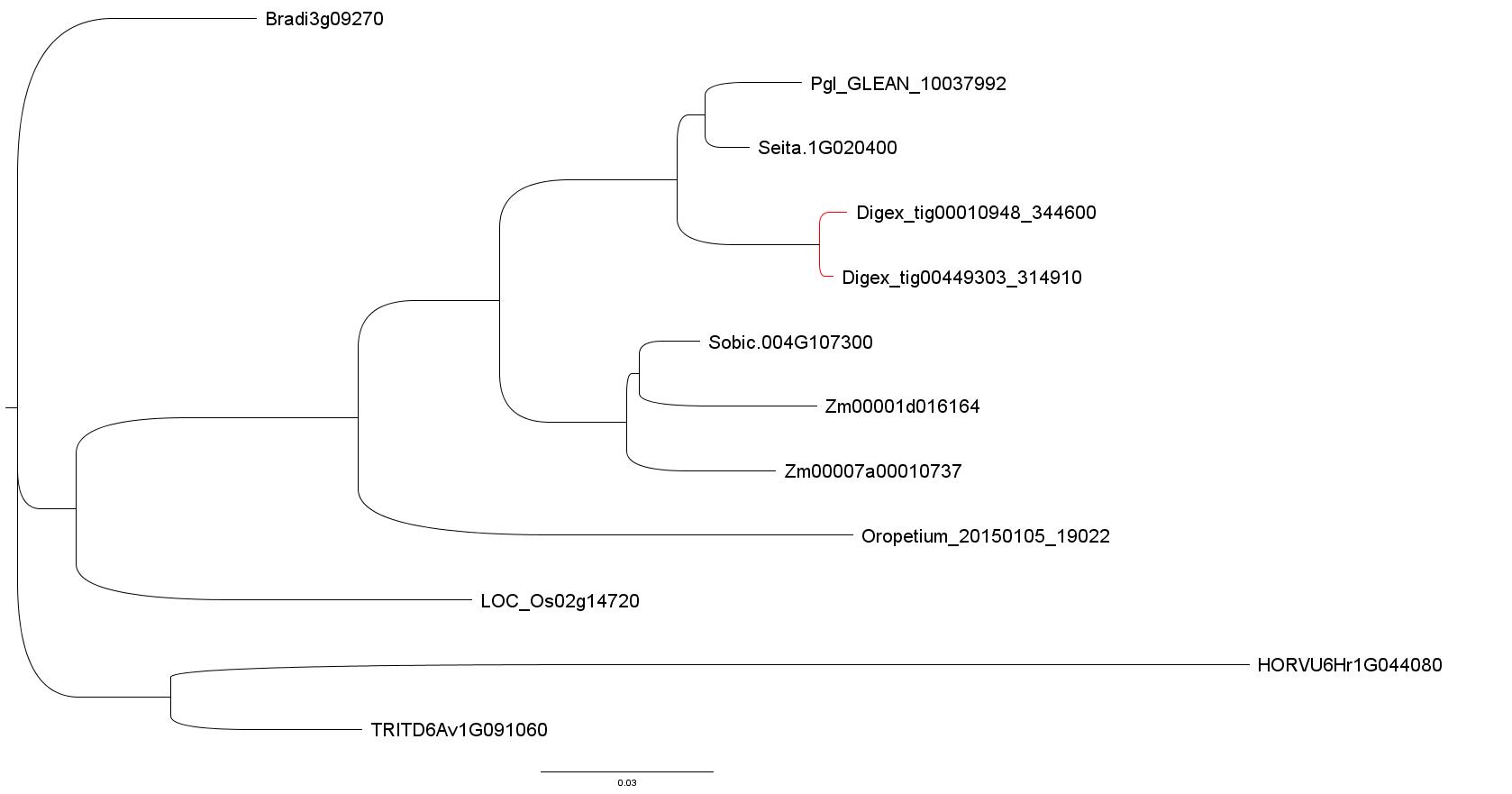


**Suppl. Figure S9.** Gene family tree for GW2-A-like genes in fonio and related species. This figure also includes the genes from two additional Pooid species, barley (Hordeum vulgare) (HORV) and wheat, Triticum turgidum (TRITD).
