# Supplementary material for: Genome sequence and genetic diversity analysis of an under-domesticated orphan crop, white fonio (Digitaria exilis)
Source: Gigascience. 2021 Mar 12;10(3):giab013. doi: 10.1093/gigascience/giab013 (PMC7953496; doi:10.1093/gigascience/giab013)
Supplement: giab013_Supplemental_Files [file giab013_supplemental_files.zip › Supplementary Methods 122020.docx]

## Paramaters for Assembly

## Settings for Canu:

canu useGrid=false –maxMemory=500G –maxThreads=48 genomeSize=1g

correctedErrorRate=0.035 --pacbio-raw pacbio_reads_1kb.fastq

## Settings for Racon:

minimap2 -t 8 asm.fa pacbio_reads.fastq > round1.paf

racon -u -t 8 pacbio_reads_1kb.fastq round1.paf asm.fa > round1.fa 2> round1.log

minimap2 -t 8 round1.fa pacbio_reads_1kb.fastq > round2.paf

racon -u -t 8 pacbio_reads_1kb.fastq round2.paf round1.fa > asm.racon.fa 2> round2.log

Settings for Arrow:

/path_to_genomicconsensus/pbmm2 align --sort -j 8 -J 4 asm.racon.fa subreads.bam asm.aligned.bam

/path_to_genomicconsensus/pbindex asm.aligned.bam

/path_to_genomicconsensus/variantCaller -j12 --algorithm=arrow -r asm.racon.fa -o asm.arrow.fasta asm.aligned.bam

Settings for Pilon:

java -jar trimmomatic-0.33.jar PE -threads 2 -basein IlluminaPE_001.fastq.gz -baseout IlluminaPE ILLUMINACLIP:./adapter.fa:2:30:20:8:true LEADING:3 TRAILING:3 SLIDINGWINDOW:4:15 MINLEN:36

bowtie2 -p 4 -x asm.arrow.fasta -1 IlluminaPE_1P -2 IlluminaPE_2P -S IlluminaPE_paired.sam

bowtie2 -p 4 -x asm.arrow.fasta -1 IlluminaPE_1U -2 IlluminaPE_2U -S IlluminaPE_unpaired.sam

samtools view -@ 2 -bh IlluminaPE_paired.sam | samtools sort -@ 2 -o IlluminaPE_paired.sorted.bam -

samtools view -@ 2 -bh IlluminaPE_unpaired.sam | samtools sort -@ 2 -o IlluminaPE_unpaired.sorted.bam -

java -Xmx400G -jar /software/pilon/1.23/static/pilon-1.23.jar –genome asm.arrow.fasta --frags IlluminaPE_paired.sorted.bam --unpaired IlluminaPE_unpaired.sorted.bam --output asm.pilon --outdir ../ --changes --vcf --threads 10

## Settings for Maker round1 (maker_opts.exe) file:

Genome=<PATH/TO/GENOME/FASTA

organism_type=eukaryotic #eukaryotic or prokaryotic. Default is eukaryotic

#-----Re-annotation Using MAKER Derived GFF3

maker_gff= #MAKER derived GFF3 file

est_pass=1 #use ESTs in maker_gff: 1 = yes, 0 = no

altest_pass=1 #use alternate organism ESTs in maker_gff: 1 = yes, 0 = no

protein_pass=1 #use protein alignments in maker_gff: 1 = yes, 0 = no

rm_pass=1 #use repeats in maker_gff: 1 = yes, 0 = no

model_pass=1 #use gene models in maker_gff: 1 = yes, 0 = no

pred_pass=1 #use ab-initio predictions in maker_gff: 1 = yes, 0 = no

other_pass=1 #passthrough anyything else in maker_gff: 1 = yes, 0 = no

#-----EST Evidence (for best results provide a file for at least one)

est_gff=</PATH/TO/EST/DATA/GFF3>

#-----Protein Homology Evidence (for best results provide a file for at least one)

protein=<PATH/TO/PROTEOME/SETARIA_SORGHUM/FASTA> #protein sequence file in fasta format (i.e. from mutiple oransisms)

#-----Repeat Masking (leave values blank to skip repeat masking)

model_org=all #select a model organism for RepBase masking in RepeatMasker

rmlib=<PATH/TO/REPET/LIBRARY/FASTA> #provide an organism specific repeat library in fasta format for RepeatMasker

repeat_protein=<PATH/TO/TRANSPOSABLE/ELEMENTS/INSTALLED/WITH/MAKER> #provide a fasta file of transposable element proteins for RepeatRunner

prok_rm=0 #forces MAKER to repeatmask prokaryotes (no reason to change this), 1 = yes, 0 = no

softmask=1 #use soft-masking rather than hard-masking in BLAST (i.e. seg and dust filtering)

#-----Gene Prediction

est2genome=1 #infer gene predictions directly from ESTs, 1 = yes, 0 = no

protein2genome=1 #infer predictions from protein homology, 1 = yes, 0 = no

trna=0 #find tRNAs with tRNAscan, 1 = yes, 0 = no

snoscan_rrna= #rRNA file to have Snoscan find snoRNAs

unmask=0 #also run ab-initio prediction programs on unmasked sequence, 1 = yes, 0 = no

#-----External Application Behavior Options

alt_peptide=C #amino acid used to replace non-standard amino acids in BLAST databases

cpus=1 #max number of cpus to use in BLAST and RepeatMasker (not for MPI, leave 1 when using MPI)

#-----MAKER Behavior Options

max_dna_len=100000 #length for dividing up contigs into chunks (increases/decreases memory usage)

min_contig=100 #skip genome contigs below this length (under 10kb are often useless)

pred_flank=200 #flank for extending evidence clusters sent to gene predictors

pred_stats=0 #report AED and QI statistics for all predictions as well as models

AED_threshold=1 #Maximum Annotation Edit Distance allowed (bound by 0 and 1)

min_protein=0 #require at least this many amino acids in predicted proteins

alt_splice=0 #Take extra steps to try and find alternative splicing, 1 = yes, 0 = no

always_complete=0 #extra steps to force start and stop codons, 1 = yes, 0 = no

map_forward=0 #map names and attributes forward from old GFF3 genes, 1 = yes, 0 = no

keep_preds=0 #Concordance threshold to add unsupported gene prediction (bound by 0 and 1)

split_hit=10000 #length for the splitting of hits (expected max intron size for evidence alignments)

single_exon=0 #consider single exon EST evidence when generating annotations, 1 = yes, 0 = no

single_length=250 #min length required for single exon ESTs if 'single_exon is enabled'

correct_est_fusion=0 #limits use of ESTs in annotation to avoid fusion genes

tries=2 #number of times to try a contig if there is a failure for some reason

clean_try=0 #remove all data from previous run before retrying, 1 = yes, 0 = no

clean_up=0 #removes theVoid directory with individual analysis files, 1 = yes, 0 = no

TMP=/data #specify a directory other than the system default temporary directory for temporary files

## Settings for Maker round2 (maker_opts.exe) file:

-----Genome (these are always required)

Genome=</PATH/TO/GENOME/FASTA>

#-----Re-annotation Using MAKER Derived GFF3

maker_gff=<PATH/TO/GFF3_FROM_ROUND1/GFF3>

est_pass=1 #use ESTs in maker_gff: 1 = yes, 0 = no

altest_pass=1 #use alternate organism ESTs in maker_gff: 1 = yes, 0 = no

protein_pass=1 #use protein alignments in maker_gff: 1 = yes, 0 = no

rm_pass=1 #use repeats in maker_gff: 1 = yes, 0 = no

model_pass=1 #use gene models in maker_gff: 1 = yes, 0 = no

pred_pass=1 #use ab-initio predictions in maker_gff: 1 = yes, 0 = no

other_pass=1 #passthrough anyything else in maker_gff: 1 = yes, 0 = no

#-----Repeat Masking (leave values blank to skip repeat masking)

model_org=#select a model organism for RepBase masking in RepeatMasker

rmlib=#provide an organism specific repeat library in fasta format for RepeatMasker

repeat_protein= #provide a fasta file of transposable element proteins for RepeatRunner

rm_gff=#pre-identified repeat elements from an external GFF3 file

prok_rm=0 #forces MAKER to repeatmask prokaryotes (no reason to change this), 1 = yes, 0 = no

softmask=1 #use soft-masking rather than hard-masking in BLAST (i.e. seg and dust filtering)

#-----Gene Prediction

Snaphmm=</PATH/TO/SNAP/FROM_ROUND1/pyu.hmm >#SNAP HMM file

gmhmm= #GeneMark HMM file

augustus_species=</PATH/TO/SPECIES/SPECIFC/AUGUSTUS/PARAMETERS/FROM_BUSCO>#Augustus gene prediction species model

fgenesh_par_file= #FGENESH parameter file

pred_gff= #ab-initio predictions from an external GFF3 file

model_gff= #annotated gene models from an external GFF3 file (annotation pass-through)

est2genome=0 #infer gene predictions directly from ESTs, 1 = yes, 0 = no

protein2genome=0 #infer predictions from protein homology, 1 = yes, 0 = no

trna=0 #find tRNAs with tRNAscan, 1 = yes, 0 = no

snoscan_rrna= #rRNA file to have Snoscan find snoRNAs

unmask=0 #also run ab-initio prediction programs on unmasked sequence, 1 = yes, 0 = no

#-----Other Annotation Feature Types (features MAKER doesn't recognize)

other_gff= #extra features to pass-through to final MAKER generated GFF3 file

#-----External Application Behavior Options

alt_peptide=C #amino acid used to replace non-standard amino acids in BLAST databases

cpus=1 #max number of cpus to use in BLAST and RepeatMasker (not for MPI, leave 1 when using MPI)

#-----MAKER Behavior Options

max_dna_len=100000 #length for dividing up contigs into chunks (increases/decreases memory usage)

min_contig=100 #skip genome contigs below this length (under 10kb are often useless)

pred_flank=200 #flank for extending evidence clusters sent to gene predictors

pred_stats=0 #report AED and QI statistics for all predictions as well as models

AED_threshold=1 #Maximum Annotation Edit Distance allowed (bound by 0 and 1)

min_protein=0 #require at least this many amino acids in predicted proteins

alt_splice=0#Take extra steps to try and find alternative splicing, 1 = yes, 0 = no

always_complete=0 #extra steps to force start and stop codons, 1 = yes, 0 = no

map_forward=0 #map names and attributes forward from old GFF3 genes, 1 = yes, 0 = no

keep_preds=0 #Concordance threshold to add unsupported gene prediction (bound by 0 and 1)

split_hit=10000 #length for the splitting of hits (expected max intron size for evidence alignments)

single_exon=0 #consider single exon EST evidence when generating annotations, 1 = yes, 0 = no

single_length=250 #min length required for single exon ESTs if 'single_exon is enabled'

correct_est_fusion=0 #limits use of ESTs in annotation to avoid fusion genes

tries=2 #number of times to try a contig if there is a failure for some reason

clean_try=0 #remove all data from previous run before retrying, 1 = yes, 0 = no

clean_up=0 #removes theVoid directory with individual analysis files, 1 = yes, 0 = no

TMP=/data #specify a directory other than the system default temporary directory for temporary files

## Maker settings for Round3:

#-----Genome (these are always required)

genome=<PATH/TO/GENOME/FASTA> #genome sequence (fasta file or fasta embeded in GFF3 file)

#-----Re-annotation Using MAKER Derived GFF3

maker_gff=<PATH_TO/GFF#_FILE_FROM_ROUND2/GFF3> #MAKER derived GFF3 file

est_pass=1 #use ESTs in maker_gff: 1 = yes, 0 = no

altest_pass=1 #use alternate organism ESTs in maker_gff: 1 = yes, 0 = no

protein_pass=1 #use protein alignments in maker_gff: 1 = yes, 0 = no

rm_pass=1 #use repeats in maker_gff: 1 = yes, 0 = no

model_pass=1 #use gene models in maker_gff: 1 = yes, 0 = no

pred_pass=1 #use ab-initio predictions in maker_gff: 1 = yes, 0 = no

other_pass=1 #passthrough anyything else in maker_gff: 1 = yes, 0 = no

#-----Repeat Masking (leave values blank to skip repeat masking)

model_org=#select a model organism for RepBase masking in RepeatMasker

rmlib=#provide an organism specific repeat library in fasta format for RepeatMasker

repeat_protein= #provide a fasta file of transposable element proteins for RepeatRunner

rm_gff=#pre-identified repeat elements from an external GFF3 file

prok_rm=0 #forces MAKER to repeatmask prokaryotes (no reason to change this), 1 = yes, 0 = no

softmask=1 #use soft-masking rather than hard-masking in BLAST (i.e. seg and dust filtering)

#-----Gene Prediction

snaphmm= #SNAP HMM file

gmhmm=<PATH/TO/GENEMARK_FILE/gmhmm.mod> #GeneMark HMM file

augustus_species=#Augustus gene prediction species model

fgenesh_par_file= #FGENESH parameter file

pred_gff= #ab-initio predictions from an external GFF3 file

model_gff= #annotated gene models from an external GFF3 file (annotation pass-through)

est2genome=0 #infer gene predictions directly from ESTs, 1 = yes, 0 = no

protein2genome=0 #infer predictions from protein homology, 1 = yes, 0 = no

trna=0 #find tRNAs with tRNAscan, 1 = yes, 0 = no

snoscan_rrna= #rRNA file to have Snoscan find snoRNAs

unmask=0 #also run ab-initio prediction programs on unmasked sequence, 1 = yes, 0 = no

#-----Other Annotation Feature Types (features MAKER doesn't recognize)

other_gff= #extra features to pass-through to final MAKER generated GFF3 file

#-----External Application Behavior Options

alt_peptide=C #amino acid used to replace non-standard amino acids in BLAST databases

cpus=1 #max number of cpus to use in BLAST and RepeatMasker (not for MPI, leave 1 when using MPI)

#-----MAKER Behavior Options

max_dna_len=100000 #length for dividing up contigs into chunks (increases/decreases memory usage)

min_contig=100 #skip genome contigs below this length (under 10kb are often useless)

pred_flank=200 #flank for extending evidence clusters sent to gene predictors

pred_stats=0 #report AED and QI statistics for all predictions as well as models

AED_threshold=1 #Maximum Annotation Edit Distance allowed (bound by 0 and 1)

min_protein=0 #require at least this many amino acids in predicted proteins

alt_splice=0 #Take extra steps to try and find alternative splicing, 1 = yes, 0 = no

always_complete=0 #extra steps to force start and stop codons, 1 = yes, 0 = no

map_forward=0 #map names and attributes forward from old GFF3 genes, 1 = yes, 0 = no

keep_preds=0 #Concordance threshold to add unsupported gene prediction (bound by 0 and 1)

split_hit=10000 #length for the splitting of hits (expected max intron size for evidence alignments)

single_exon=0 #consider single exon EST evidence when generating annotations, 1 = yes, 0 = no

single_length=250 #min length required for single exon ESTs if 'single_exon is enabled'

correct_est_fusion=0 #limits use of ESTs in annotation to avoid fusion genes

tries=2 #number of times to try a contig if there is a failure for some reason

clean_try=0 #remove all data from previous run before retrying, 1 = yes, 0 = no

clean_up=0 #removes theVoid directory with individual analysis files, 1 = yes, 0 = no

TMP=/data #specify a directory other than the system default temporary directory for temporary files

## Genetic diversity analysis

A brief summary of software and settings for genetic diversity analysis is presented below. For the full pipeline, please see the Github repository at <https://github.com/wallacelab/paper-fonio-diversity-2020>, which contains the complete pipeline, all support scripts, and instructions for recreating the Conda environment used for analysis.

Principal coordinates were calculated by using classical multidimensional scaling (R function cmdscale()) on a genetic distance matrix calculated in TASSEL (option –distanceMatrix). The same distance matrix was used to create the dendrogram by neighbor-joining (function nj()) with the R package app v5.3 [87]. Accessions were plotted geographically using the R package ggmap v3.0.0 [85]. Additional software used in this analysis included samtools v0.1.19-96b5f2294a [88], conda 4.8.3 [89], PLINK v1.90b5.2 [90] and the R packages argparse v2.0.1 [91], ggplot2 v3.2.1 [92], gridExtra v2.3 [93], and RColorBrewer v1.1.2 [94].

### SNP Calling

Quality-filtered sequencing data from Data2Bio was aligned to the genome sequence using

GSNAP v2020-04-08 [78] using default parameters. SNPs were called using the bcftools mpileup command v1.9 [79] with max-depth set to 1000 and minimum base quality set to 20. Only bialleleic SNPs were kept. These raw SNPs were then filtered using TASSEL v5.2.40 [80], custom R scripts with R v3.5.1 [81], and bcftools to include only sites with ≤25% heterozygosity, ≤500 total read depth, ≤60% missing data, and ≥2.5% minor allele frequency.

### Population Structure

Population substructure was determined with fastStructure v1.0 [82], testing from 1 to 10 population clusters and identifying the optimal number with the included chooseK.py program. Genetic principal coordinates were calculated by using classical multidimensional scaling (R function cmdscale()) on a genetic distance matrix calculated in TASSEL (option –distanceMatrix). The same distance matrix was used to create the dendrogram by neighbor-joining (function nj()) with the R package ape v5.3 [83]. Accessions were plotted geographically using the R package ggmap v3.0.0 [81].
