# Supplementary material for: Genome sequence and genetic diversity analysis of an under-domesticated orphan crop, white fonio (Digitaria exilis)
Source: Gigascience. 2021 Mar 12;10(3):giab013. doi: 10.1093/gigascience/giab013 (PMC7953496; doi:10.1093/gigascience/giab013)
Supplement: giab013_Response_to_Reviewer_Comments_Original_Submission [file giab013_response_to_reviewer_comments_original_submission.pdf]

Author's Response To Reviewer Comments

Close

See enclosed file under Personal Cover-Response to Review and Manuscript R1 tracked changes.

Close
