# Supplementary material for: Genome sequence and genetic diversity analysis of an under-domesticated orphan crop, white fonio (Digitaria exilis)
Source: Gigascience. 2021 Mar 12;10(3):giab013. doi: 10.1093/gigascience/giab013 (PMC7953496; doi:10.1093/gigascience/giab013)
Supplement: giab013_Reviewer_1_Report_Original_Submission — Xupo Ding -- 8/24/2020 Reviewed [file giab013_reviewer_1_report_original_submission.pdf]

Reviewer Report

Title: Genome sequence and genetic diversity analysis of an under-domesticated orphan crop, white fonio (Digitaria exilis)

Version: Original Submission **Date:** 8/24/2020

Reviewer name: Xupo Ding

Reviewer Comments to Author:

Although the species mentioned in this article is minor cereal crop, but the D.exilis can improve soil utilization rate via filling the dietary gap before other crops maturation in West Africa and this special species could acclimatized the exceptional habitats, such as drought, hot or low fertility, and the shorter growing period resulting in it might be the potential model germplasm for genetic improvement of cereal crops. This manuscript provided a high-quality assembly with a well annotation of D.exilis. The population genetics analysis produced very interesting results. In addition, it is worth promotion that authors shared the codes files on GitHub leading us to reproduce the results in this manuscript. I would like to recommend this manuscript publish on GigaScience after revised some concerns.

P.S. Consideration of the original manuscript submitted without the line number (please add in the revision), the concerns would suggest on the basis of section.

1. The original manuscript seemed to a well oral draft for speaker, especially in the sections of background, plant material and conclusions.
2. In the section of Plant material and nucleic preparation, please provide the original source of fonio seeds and its latitude-longitude. The contents and their proportions of standard potting soil should be indicated.
3. In the logically, estimation of genome and heterozygosity with illumina reads were before assembly of PacBio reads and polished with illumine reads. Please consider, this is not must revised if it were not necessary.
4. Repeat annotation and TE properties are continuous. In the section of TE properties, average size of Gypsy is the double of Copia, why?
5. For more intuitively, please add the most recent common ancestor and predicted divergence time or confidence interval beside the node in Fig1D.
6. I noticed that KAUST has been upload their genome of D.exilis on NCBI with the BioProject number: PRJEB36539. The quality of assembly sequence in this manuscript is much higher than theirs. Comparing or mentioning their assembly will look fair and highlight the higher quality assembly of the genome you present here.
7. The candidate domestication genes were well aligned and discussed. But in the abstract, resilience in hot, dry and low fertility environments of D.exilis were highlighted. Have you found special gene families related with these physiological features in your analysis? Excellent resources of resistance gene or TFs are also important for genetic improvement of other cereal crops. Expansion and contraction of gene family might provide some preliminary clues.

8. RRID numbers were not contained in this original manuscript.

Level of Interest

Please indicate how interesting you found the manuscript: Choose an item.

Quality of Written English

Please indicate the quality of language in the manuscript: Choose an item.

Declaration of Competing Interests

Please complete a declaration of competing interests, considering the following questions:

- Have you in the past five years received reimbursements, fees, funding, or salary from an organisation that may in any way gain or lose financially from the publication of this manuscript, either now or in the future?
- Do you hold any stocks or shares in an organisation that may in any way gain or lose financially from the publication of this manuscript, either now or in the future?
- Do you hold or are you currently applying for any patents relating to the content of the manuscript?
- Have you received reimbursements, fees, funding, or salary from an organization that holds or has applied for patents relating to the content of the manuscript?
- Do you have any other financial competing interests?
- Do you have any non-financial competing interests in relation to this paper?

If you can answer no to all of the above, write 'I declare that I have no competing interests' below. If your reply is yes to any, please give details below.

I declare that I have no competing interests

I agree to the open peer review policy of the journal. I understand that my name will be included on my report to the authors and, if the manuscript is accepted for publication, my named report including any attachments I upload will be posted on the website along with the authors' responses. I agree for my report to be made available under an Open Access Creative Commons CC-BY license (http://creativecommons.org/licenses/by/4.0/). I understand that any comments which I do not wish to be included in my named report can be included as confidential comments to the editors, which will not be published.

Choose an item.

To further support our reviewers, we have joined with Publons, where you can gain additional credit to further highlight your hard work (see: https://publons.com/journal/530/gigascience). On publication of this paper, your review will be automatically added to Publons, you can then choose whether or not to claim your Publons credit. I understand this statement.

Yes Choose an item.
