# Supplementary material for: Genome sequence and genetic diversity analysis of an under-domesticated orphan crop, white fonio (Digitaria exilis)
Source: Gigascience. 2021 Mar 12;10(3):giab013. doi: 10.1093/gigascience/giab013 (PMC7953496; doi:10.1093/gigascience/giab013)
Supplement: giab013_Reviewer_3_Report_Original_Submission — Gincy P. Thottathil -- 9/13/2020 Reviewed [file giab013_reviewer_3_report_original_submission.pdf]

Reviewer Report

Title: Genome sequence and genetic diversity analysis of an under-domesticated orphan crop, white fonio (Digitaria exilis)

Version: Original Submission **Date:** 9/13/2020

Reviewer name: Gincy P. Thottathil

Reviewer Comments to Author:

The manuscript entitled "Genome sequence and genetic diversity analysis of an under- domesticated orphan crop, white fonio (Digitaria exilis)" is an excellent report on the exemplary research done by the authors. Considering the nutritional importance and the lack of adequate genetic information, fonio needs more research attention. Appreciate the initiative taken by the authors which would definitely be helpful in future breeding programs. But, a more complete assembly of Digitaria exilis genome has already been published a few days back.
Abrouk, M., Ahmed, H. I., Cubry, P., Å imonÃ-kovÃ¡, D., Cauet, S., Pailles, Y., ... & Zekraoui, L. (2020). Fonio millet genome unlocks African orphan crop diversity for agriculture in a changing climate. Nature Communications, 11(1), 1-13.
However, considering the additional analysis and information provided in this manuscript, I strongly recommend the publication of this manuscript.

1. Two sequencing platforms were used in the study; PacBio and Illumina. Even though the assembly almost covers the estimated genome size, it is presented as 3333 contigs. What is the difficulty faced by the authors to construct it into chromosome level? Small genome size as well as the availability of long reads could make it more feasible to construct the chromosomes. Also, only 88% of the RNAseq reads could be aligned to genome, which shows the incompleteness of the assembly. How do the authors justify this?
2. Line no. 83
"is some transcript sequence data [13] at NCBI."
Line No. 164
"Illumina RNA sequencing data (paired-end 100 bp) of Digitaria exilis [13] were downloaded".
Here the reference cited is wrong.
3. 67855 protein coding gene were identified from the assembly. This is quite a large number compared to other related plants. However, it is expected due to the allotetraploid nature of the plant and 4.3% single nucleotide variation was observed in between the sub- genomes. All analyzed domestication genes are duplicated in fonio compared to other related plants. Apart from the expected doubling, is there an expansion in any particular gene family?
4. Is there any gene unique to either of the sub- genomes?
5. Please provide legends for Fig. S1- A and B.

Level of Interest

Please indicate how interesting you found the manuscript: Choose an item.

Quality of Written English

Please indicate the quality of language in the manuscript: Choose an item.

Declaration of Competing Interests

Please complete a declaration of competing interests, considering the following questions:

- Have you in the past five years received reimbursements, fees, funding, or salary from an organisation that may in any way gain or lose financially from the publication of this manuscript, either now or in the future?
- Do you hold any stocks or shares in an organisation that may in any way gain or lose financially from the publication of this manuscript, either now or in the future?
- Do you hold or are you currently applying for any patents relating to the content of the manuscript?
- Have you received reimbursements, fees, funding, or salary from an organization that holds or has applied for patents relating to the content of the manuscript?
- Do you have any other financial competing interests?
- Do you have any non-financial competing interests in relation to this paper?

If you can answer no to all of the above, write 'I declare that I have no competing interests' below. If your reply is yes to any, please give details below.

I declare that I have no competing interests

I agree to the open peer review policy of the journal. I understand that my name will be included on my report to the authors and, if the manuscript is accepted for publication, my named report including any attachments I upload will be posted on the website along with the authors' responses. I agree for my report to be made available under an Open Access Creative Commons CC-BY license (http://creativecommons.org/licenses/by/4.0/). I understand that any comments which I do not wish to be included in my named report can be included as confidential comments to the editors, which will not be published.

Choose an item.

To further support our reviewers, we have joined with Publons, where you can gain additional credit to further highlight your hard work (see: https://publons.com/journal/530/gigascience). On publication of this paper, your review will be automatically added to Publons, you can then choose whether or not to claim your Publons credit. I understand this statement.

Yes Choose an item.
